# Identification and motif analyses of candidate nonreceptor olfactory genes of *Dendroctonus adjunctus* Blandford (Coleoptera: Curculionidae) from the head transcriptome

**DOI:** 10.1038/s41598-020-77144-5

**Published:** 2020-11-26

**Authors:** Brenda Torres-Huerta, Obdulia L. Segura-León, Marco A. Aragón-Magadan, Héctor González-Hernández

**Affiliations:** Entomology and Acarology Program, Colegio de Postgraduados Campus Montecillo, Mexico-Texcoco, km. 36.5, C. P. 56230 Montecillo, State of Mexico Mexico

**Keywords:** Animal behaviour, Entomology, Molecular evolution, Phylogenetics, Speciation, Gene ontology, Phylogeny, Protein analysis, Protein function predictions, Sequence annotation, Behavioural ecology, Forestry, Computational biology and bioinformatics, Ecology, Evolution, Zoology

## Abstract

The round-headed pine beetle *Dendroctonus adjunctus*, whose dispersion and colonization behaviors are linked to a communication system mediated by semiochemicals, is one of the five most critical primary pests in forest ecosystems in Mexico. This study provides the first head transcriptome analysis of *D. adjunctus* and the identification of the nonreceptor olfactory genes involved in the perception of odors. De novo assembly yielded 44,420 unigenes, and GO annotations were similar to those of antennal transcriptomes of other beetle species, which reflect metabolic processes related to smell and signal transduction. A total of 36 new transcripts of nonreceptor olfactory genes were identified, of which 27 encode OBPs, 7 encode CSPs, and 2 encode SNMP candidates, which were subsequently compared to homologous proteins from other bark beetles and Coleoptera species by searching for sequence motifs and performing phylogenetic analyses. Our study provides information on genes encoding nonreceptor proteins in *D. adjunctus* and broadens the knowledge of olfactory genes in Coleoptera and bark beetle species, and will help to understand colonization and aggregation behaviors for the development of tools that complement management strategies.

## Introduction

In Mexico, there are five species of bark beetles classified as primary pests, and the most important in high-altitude regions is *Dendroctonus adjunctus*, which is considered the primary pest in pine forests above 2,800 m asl^1,2^. Atypical epidemic outbreaks of bark beetles have occurred in the last decade in several states of Mexico, and in the last five years, there have been intense attacks in the Sierra Madre Oriental, the Transversal Volcanic Axis, and the Sierra Madre del Sur^3,4^. These beetles have developed a complex communication system mediated by semiochemicals essential in host colonization and mating^5,6^; this system has been used as a basis for developing eco-friendly management strategies^7,8^. However, due to their global ecological and economic impact, there is a great challenge in generating knowledge about the physiology and molecular biology of the perception of semiochemicals and signal processing.

Antennae are the primary sensory organs of insects^9–11^; olfactory processing includes different perireceptor events^12,13^, where a set of receptors and nonreceptor olfactory proteins are involved. The olfactory receptors are transmembrane proteins responsible for recognizing and discriminating different semiochemicals^14–16^. This group includes odorant receptors (ORs), ionotropic receptors (IRs), and gustatory receptors (GRs) proteins. The nonreceptors involve three main types of proteins: those that solubilize and transport small hydrophobic compounds, which includes odorant-binding proteins (OBPs) and chemosensory proteins (CSPs)^17^; and sensory neuron membrane proteins (SNMPs) which is a family of transmembrane proteins with a role in the insect olfactory system^18^.

OBPs and CSPs represent a heterogeneous group of small (12–18 kDa) soluble polypeptides that have a hydrophobic binding cavity made of α-helicoidal domains folded in two different patterns^19,20^. OBPs form three interlocking disulfide bridges and are typically divided into five subclasses according to the number of conserved Cys residues in the primary structure. Members of the Classic subclass with six-Cys pattern include pheromone-binding proteins (PBPs) and general odorant-binding proteins (GOBPs); C-minus subclass members lack two of those Cys residues; Plus-C members have 4–6 additional Cys residues and a specific proline; and members of another classes are dimers with two conserved 6-Cys patterns, atypical 9–10 Cys residues and a long C-terminal region^21^. In contrast, the CSPs have a motif of four conserved Cys residues that form two interhelical disulfide bonds and, unlike OBPs, form a more homogeneous group^22,23^. Initially, their functions were assumed to chemoreception mechanisms because they were characterized in the antennae and oral organs^24^.

However, in the last decade, these proteins have been identified and characterized in nonsensory organs, suggesting other physiological functions^25^, such as detecting and releasing pheromones into the environment in specialized glands^26–28^, carrying nutrients^29,30^, and even capturing and masking toxic insecticide molecules in different parts of the body^31^.

SNMPs compose a subclass of insect genes homologous to the CD36 proteins; currently, two subfamilies (SNMP1 and SNMP2) have been described in different insect orders, such as Diptera, Lepidoptera, Hymenoptera, Orthoptera, and Coleoptera^32,33^. SNMP1 is associated with specific olfactory pheromone neuron receptors^34^, while SNMP2 has been shown to be expressed in pheromone-sensitive sensory support cells^35^ in the legs and wings of Diptera insects^36^.

The study of these proteins has sharply increased in the last decade, and their size, stability, resistance to high temperature, and proteolytic digestion make them candidates for the development of different biotechnological tools and applications^37,38^ in agriculture, such as the design of biosensors^39,40^, the development of specific chemical repellents or attractants^41,42^ and the development of interference mechanisms in host location and mating^43,44^. Additionally, the olfactory recognition of insects differs according to their environment and evolutionary history, which requires further research of additional species to obtain a better understanding of the peripheral olfactory proteins involved in perireceptor events. Therefore, we assembled the transcriptome of *D. adjunctus* heads to identify and analyze the repertoire of genes encoding candidate nonreceptor proteins involved in odor processing because knowledge of these genes and proteins has the potential for the development and application of tools in integrated pest management based on olfactory recognition processes.

## Results

### Cleaning and sequence assembly

With the BGISEQ-500 platform, an average of 96,631,854 reads (3.5 GB) with a total length of 100 bp were obtained from three libraries of male and female heads of *D. adjunctus* with a Q20 of 98.13% and GC% of 36.89. According to the cleaned read results, the libraries had a low percentage of adapters (⁓0.081%) and lacked low-quality reads. De novo assembly resulted in 71,157 transcripts with an average length of 574 bp, an N50 length of 3,126 bp and a GC percentage of 41.2%. After sorting and redundancy filtering, the numbers were reduced to 44,420 unigenes (supplementary Table S1).

### Gene ontology (GO) annotation and homology analyses

**Figure 1:**
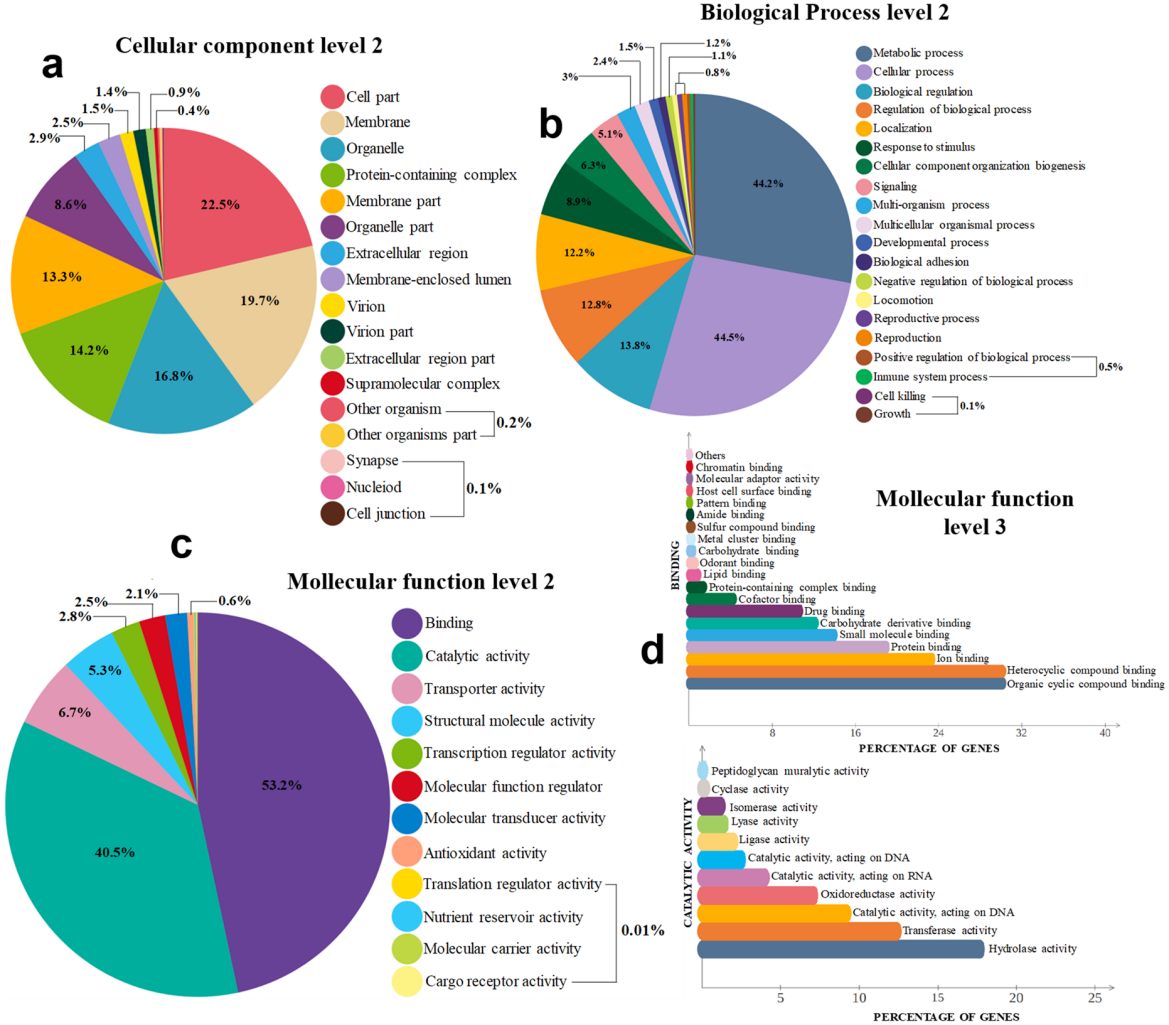
GO analysis of the *D. adjunctus* head transcriptome. Categories of (**a**) cell components, (**b**) biological processes, and (**c**) molecular functions, represented at level 2 of the GO classification in addition to level 3 (**d**) for the subcategories of binding and catalytic activity of molecular functions. The results are presented as percentages according to 13,883 transcripts with a GO assignment.

As a result of the classification of the unigenes according to the Gene Ontology terms, 31.25% of the 44,420 transcripts were classified into the three GO categories and divided into 57 functional groups (Fig. 1). Annotations associated with molecular function (MF) were the most abundant (44.66%).

The main subcategories represented in the MF category (greater than 90%) (Fig. 1c) were binding (53.2%) and catalytic activity (40.5%). In the first case, the most representative functions were binding to cyclic-heterocyclic organic compounds, ion binding, and protein binding, while hydrolase activity was one of the most abundant catalytic activities (Fig. 1d). The categories of biological processes (BPs) and cellular components (CCs) constituted 34.03% and 21.03%, respectively. For BPs (Fig. 1b), the most abundant subcategories were related to molecular (41%) and metabolic processes (44%), while among the CCs (Fig. 1a), the subcategory with most annotated transcripts was cellular parts (22%).

**Figure 2:**
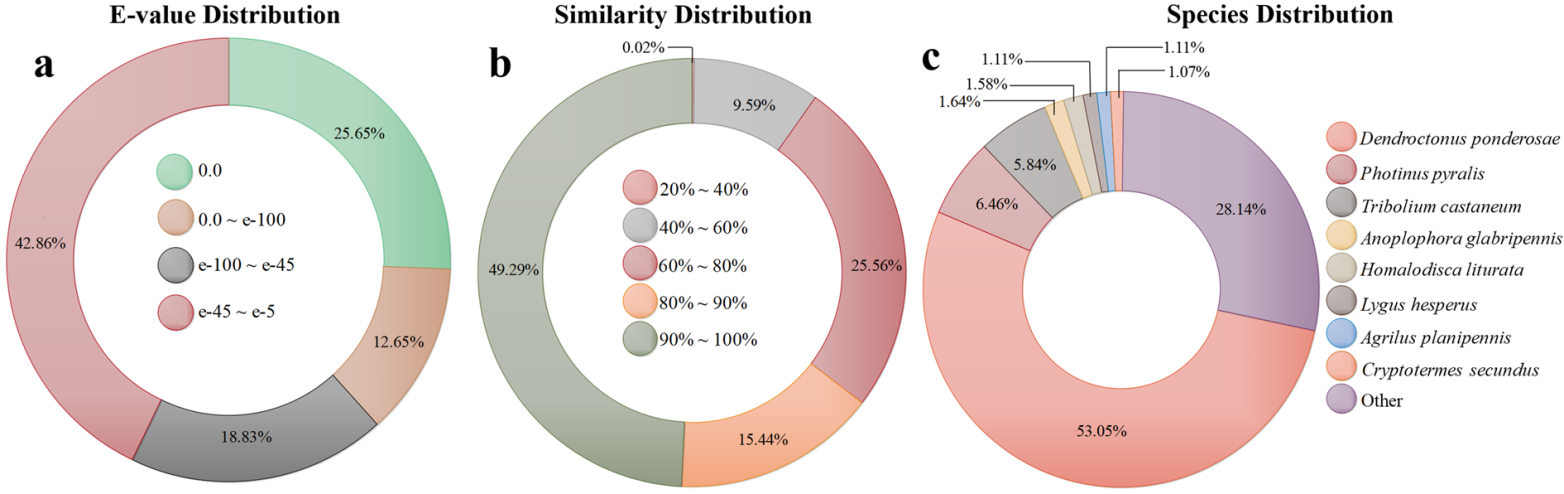
Distribution of homology results. Percentage of transcripts mapped against the UniProtKB database. (**a**) E-value distribution, (**b**) percentage similarity distribution, (**c**) species distribution.

According to a BLASTx homology analysis, a total of 16,290 unigenes were mapped against the Insecta UniProtKB database, with an E-value of 1.0e−5, of which 57.14% had matches with very high-quality values below 1.0e−45 (Fig. 2a), and 42.86% ranged from 1.0e−45 to 1.0e−5. Based on the distribution of the percentages of similarity, most of the matches had values greater than 90%; moreover, with respect to the distribution of species with similarity (Fig. 2b), more than three-quarters of the unigenes matched with those of Coleoptera species, and more than 50% displayed similarity with the genes of *D. ponderosae* (Fig. 2c), which is a closely related species belonging to the same subfamily (Curculionidae: Scolytinae).

### Odorant-binding proteins

We identified a total of 27 transcripts encoding OBPs, of which 24 had a complete open reading frame (ORF), encoding proteins with lengths ranging from 110 to 390 residues, and for all sequences, we recorded peptides with a predicted signal, except for DadjOBP15. All DadjOBPs had homologs with odorant-binding proteins of other Coleoptera species, and 21 sequences had high identities with those of Scolytinae, of which 19 had similarity values > 80% with those of *D. ponderosae* (supplementary Table S2).

Except for DadjOBP3, DadjOBP9 and DadjOBP31, all sequences were functionally annotated by searching for domains within the superfamily of insect pheromones/odorant-binding proteins (supplementary Table S3). The DadjOBPs were divided into three subclasses (supplementary Fig. S1–S3), of which 15 were classified into the classic type with the general Cys motif reported for Coleoptera (C1-X_23-44_-C2-X_3_-C3-X_36-43_-C4-X_8-12_-C5-X_8_-C6), 10 DadjOBPs were in the minus-C subclass, with the absence of cysteines C2 and C5, and only DadjOBP2 was classified as plus-C, with pattern similar to that of the classic type but with seven additional cysteines and one conserved proline at the C-terminus.

**Figure 3:**
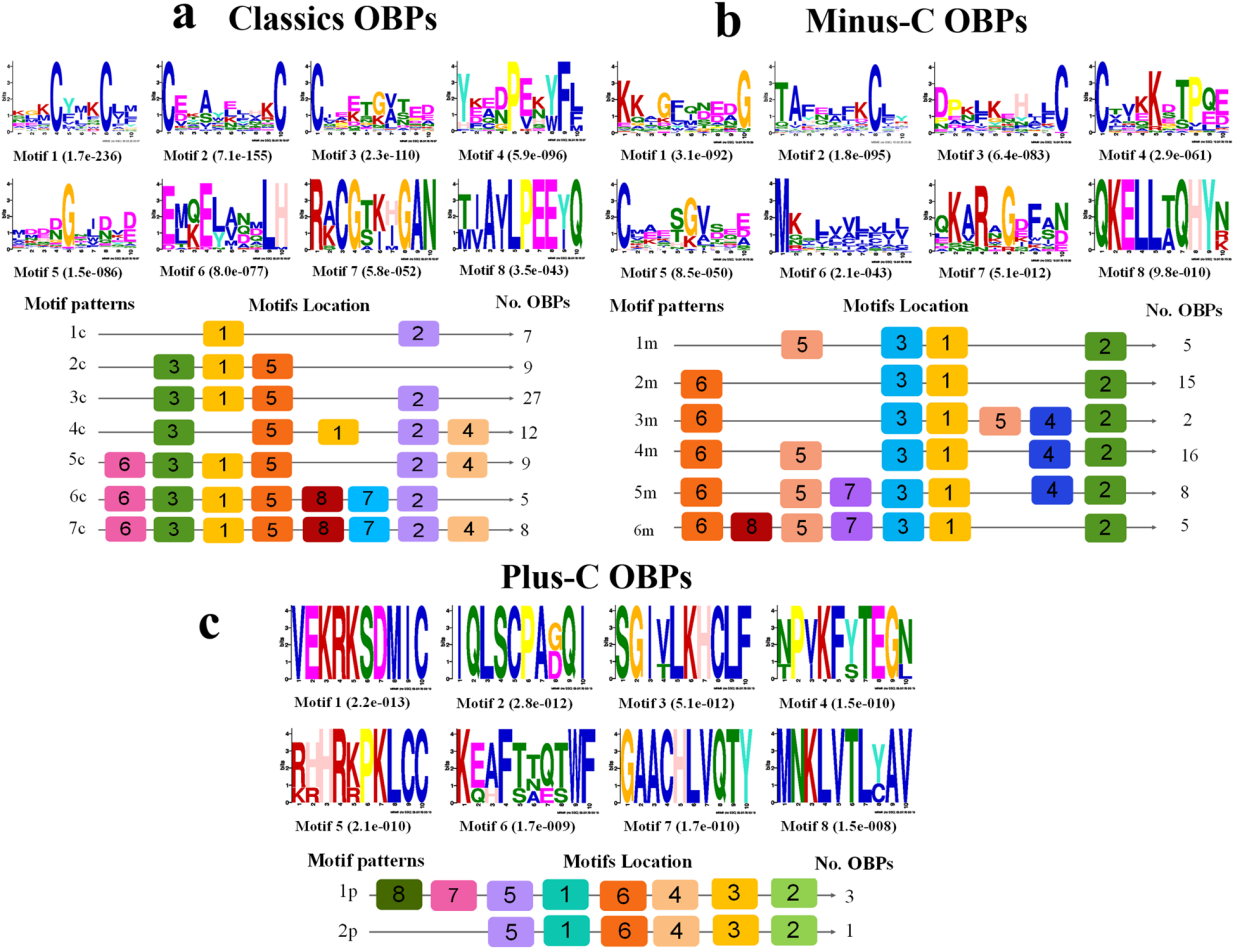
Motifs of three odorant-binding protein subclasses of *D. adjunctus* and orthologs. The sequences are displayed in three sets: 79 classic OBPs (**a**), 51 minus-C OBPs (**b**), and 4 plus-C OBPs (**c**). In the lower part, the column on the right shows the numbers of OBPs corresponding to the motif patterns that are on the left (c = classic, m = minus, p = plus), and the numbers in the boxes correspond to the different motifs shown in the upper part of the figures. The residue size is directly associated with its frequency in the alignment, and a relatively small E-value means a relatively high degree of conservation.

For the analysis of sequence motifs by MEME-suite 5.1.1, the DadjOBP sequences were separated into three sets according to their subfamily and grouped with homologous proteins of Scolytinae and *Tribolium castaneum*. For classic OBPs, we analyzed a total of 72 proteins that contained eight motifs (Fig. 3a) and that were grouped into seven distinct patterns (1c-7c). Motif 1 was presented in 100% of the proteins, while motifs 2, 3 and 5 were present in 94% of the sequences. Motifs 1, 2 and 3 correspond to three of the characteristic features of classic OBPs, in which conserved residues of cysteine (Cys) form the three disulfide bridges essential to the OBP structure. On the other hand, pattern 3c was present in 39% of proteins and included the four motifs conserved in more than 90% of classic OBPs. Patterns 5c, 6c and 7c were present in only 22 proteins and are the unique patterns that had motifs 6, 7 and 8, which have highly conserved residues among the sequences.

For the minus-C motif analysis, we used a total of 51 proteins. All the sequences presented eight motifs grouped in seven different motif patterns; motifs 3, 1, and 2 were conserved in all OBPs, and motif 6 was conserved in 90.2% of the sequences (Fig. 3b). Motifs 3 and 2 represent C2 and C4 of the general Cys profile conserved in the minus-C subclass, while motifs 5 and 4 have two Cys residues with invariable positions corresponding to C1 and C3, respectively. The most frequent motif patterns, with 60% of the sequences being 2 m and 4 m, share motifs 6-3-1-2, but the 4 m pattern has two additional motifs (5 and 4). Finally, for the plus-C subclass analysis with only four sequences of Scolytinae OBPs, we identified eight sequence motifs grouped in two patterns (Fig. 3c). Pattern 1p, which presents all the described motifs, was identified for both DadjOBP2 and DponOBP2 (2), while ItypOBP2, with the 2p pattern, lacks motifs 8 and 7 of the N-terminus.

**Figure 4:**
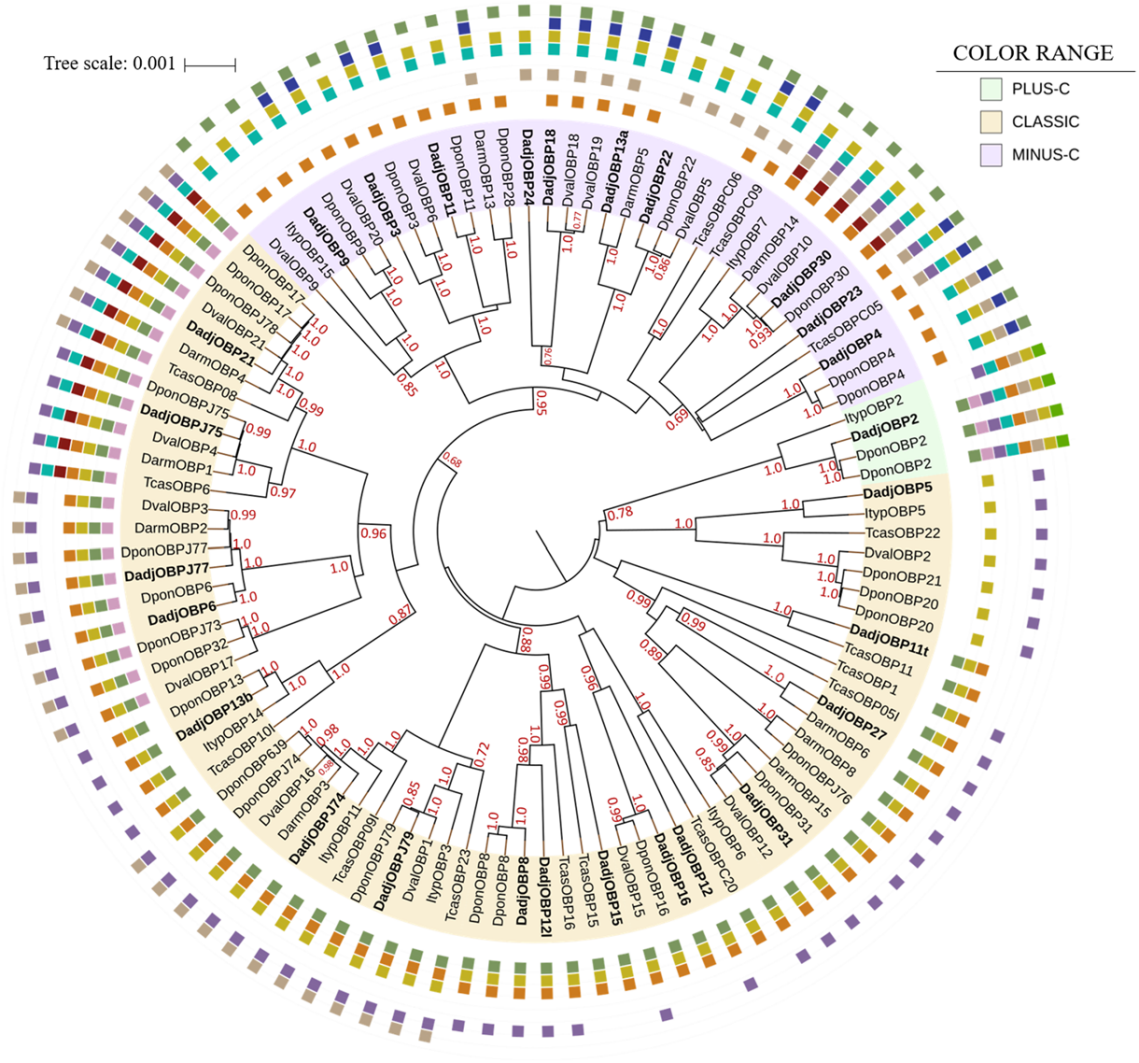
BEAST phylogenetic tree of candidate odorant-binding proteins (OBPs). Evolutionary Bayesian analysis of OBPs from *Dendroctonus adjunctus* (Dadj), *D. ponderosae* (Dpon), *D. valens* (Dval), *D. armandi* (Darm), *Ips typographus* (Ityp) and *Tribolium castaneum* (Tcas). The OBP classes displayed classic (yellow), minus-C (purple) and plus-C (green) patterns. Posterior probability values greater than 70% are presented, and the motif patterns (p < 0.0001) were obtained from MEME in the colored squares (Fig. 3). The tree was constructed via iTOL.

To understand the evolutionary relationship of the DadjOBPs with other proteins homologous to those of bark beetles and *T. castaneum*, we constructed a phylogenetic tree via Bayesian inference and included the results of the motif analysis obtained for the OBP subclasses (Fig. 4). In the phylogenetic tree, we observed the division of proteins according to the Cys profiles conserved in the three subclasses of OBPs with posterior probabilities of mostly > 95%. The classic OBPs were the most representative group, whose motif patterns 1c, 2c, 3c and 4c were the most abundant and present motifs with the main characteristics of the structural importance of this subclass. Patterns 5c, 6c and 7c clustered within an internal clade, and only these patterns contained additional motifs with highly conserved residues (6, 8 and 7).

The minus-C and plus-C OBPs grouped into two different clades related to the classic subclass and present patterns with a relatively high degree of conserved motifs in the same positions of the protein sequences (Fig. 4). The minus-C OBPs with patterns 3 m, 6 m and 7 m were grouped at the base of the phylogenetic tree, which, in contrast to the more frequent patterns, presented two additional motifs (7 and 8) and a variation in the position of motif 5. Finally, four Scolytinae OBPs grouped in the plus-C clade, where *Dendroctonus* species had the same pattern of motifs and had an amino acid identity > 90%.

### Chemosensory proteins

In the *D. adjunctus* head transcriptome, we identified seven transcripts for CSP with full ORFs that encode proteins whose length ranges from 116 to 296 residues. All CSPs had homologs with chemosensory proteins of *D. ponderosae*, with identities > 80% (supplementary Table S2), and were functionally annotated by searching for domains within the insect chemosensory and odorant-binding protein superfamily A10/Ejaculate Bulb Specific Protein 3 (supplementary Table S3).

**Figure 5:**
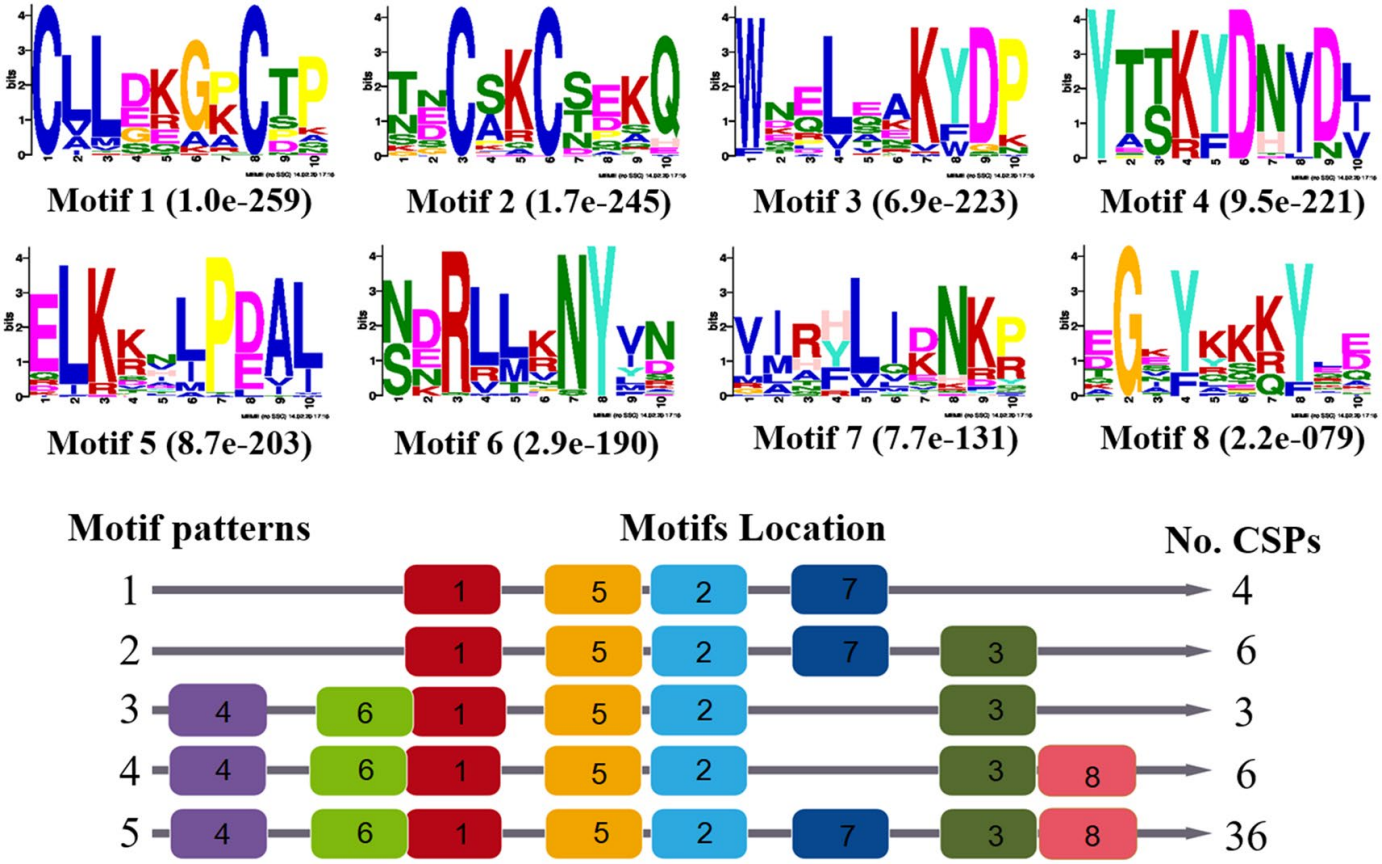
Motifs of DadjCSPs and orthologs. In the lower part are shown the motifs of CSPs; the column on the right shows the numbers of CSPs corresponding to the motif patterns, which are on the left, and the numbers in the boxes correspond to the different motifs shown in the upper part of the figure. The residue size is directly associated with its frequency in the alignment, and a relatively small E-value means a relatively high degree of conservation.

The DadjCSPs sequence had four conserved cysteines with a general pattern C1-X_6_-C2-X_18_-C3-X_2-_C4. A sequence motif analysis was performed with a set of 55 proteins that included CSPs from four species of Scolytinae and *T. castaneum*. We identified a total of eight motifs grouped into five different patterns, where motifs 1, 5 and 2 were present in 100% of the sequences; these correspond to the characteristic motifs of CSPs (Fig. 5). Motif pattern 5 was present in 65% of the CSPs and was composed of eight motifs, while the other patterns varied in their number of motifs but not in position.

**Figure 6:**
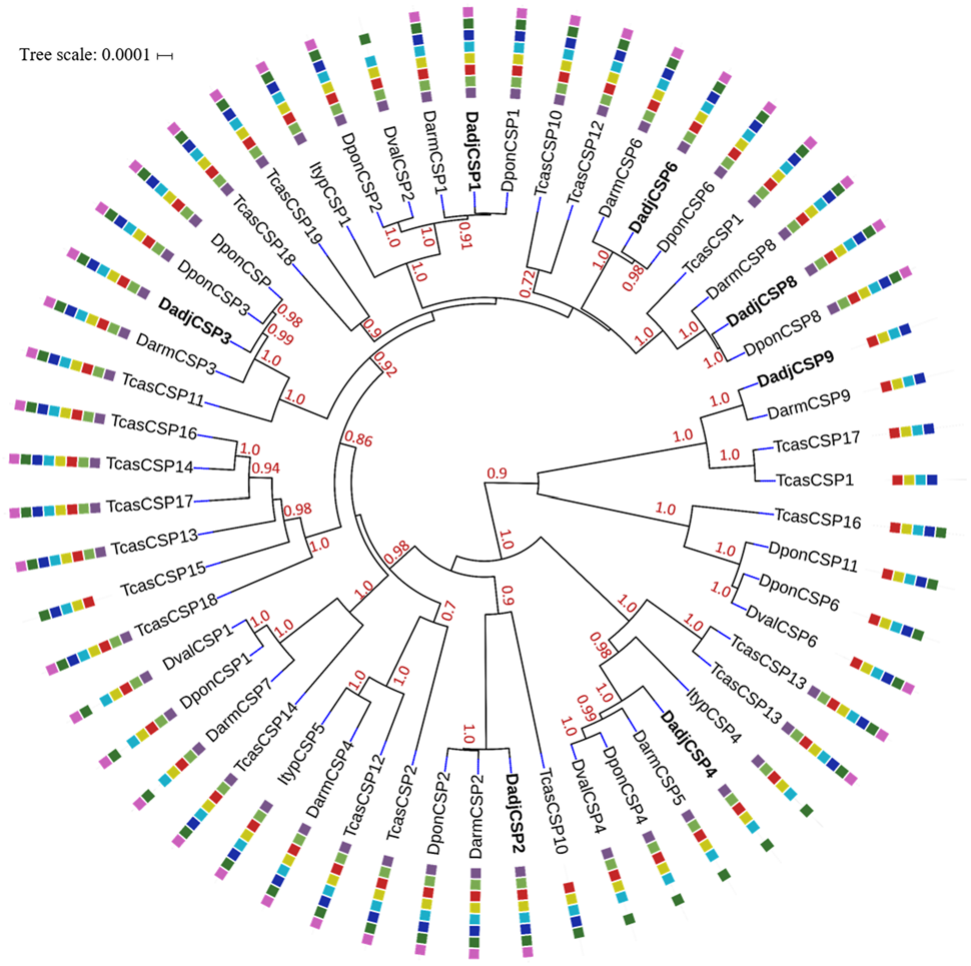
BEAST phylogenetic tree of chemosensory proteins (CSPs). Evolutionary Bayesian analysis of CSPs from *Dendroctonus adjunctus* (Dadj), *D. ponderosae* (Dpon), *D. valens* (Dval), *D. armandi* (Darm), *Ips typographus* (Ityp) and *Tribolium castaneum* (Tcas). The posterior probability values greater than 70% are displayed, and CSP motif patterns obtained from MEME are shown in the colored squares (p < 0.0001). The tree was constructed via iTOL.

Using Bayesian inference, we performed a phylogenetic analysis of DadjCSPs with homologous proteins from four Scolytinae species and *T. castaneum*, and the patterns from the motif analysis were incorporated into the tree construction. The phylogenetic tree had posterior probabilities that were mostly > 95%, and the DadjCSPs were dispersed among the different branches of the tree; they were closely related to the CSPs of *D. ponderosae* and *D. armandi* (Fig. 6). On the other hand, motif patterns 3, 4 and 5 were distributed among the branches within the same clade, and most of the patterns had the eight motifs described, while proteins with patterns 1 and 2, which lack motifs 4, 6 and 8 (except DvalCSP6), were in an external clade grouped into two clusters.

### Sensory neuron membrane proteins

We identified two transcripts for SNMPs with full ORFs in the head transcriptome of *D. adjunctus*, and for both proteins, two transmembrane helices were predicted in both the N- and C-terminal regions, with a long extracellular loop. Both DadjSNMPs were homologous to SNMP1a and SNMP of *D. ponderosae*, with an E-value of 0 and an identity > 90% (supplementary Table S2), and were functionally annotated by searching for domains within the CD36 superfamily (supplementary Table S3).

**Figure 7:**
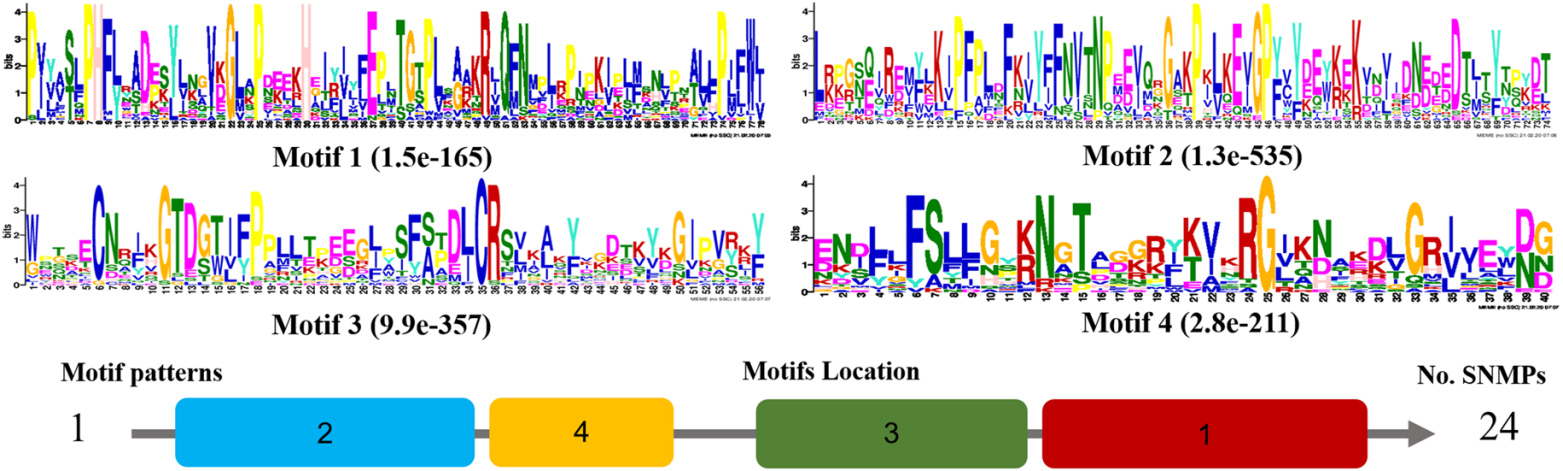
Motifs of sensory neuronal membrane proteins (SNMPs) of *D. adjunctus* and orthologous proteins. The parameters used for motif discovery were nmotif = 10, minw = 45 and maxw = 95. In the lower part, the column on the right shows the number of SNMPs corresponding to the motif patterns, which are on the left, and the numbers in the boxes correspond to the different motifs shown in the upper part of the figure. The residue size is directly associated with its frequency in the alignment, and a relatively small E-value means a relatively high degree of conservation.

**Figure 8:**
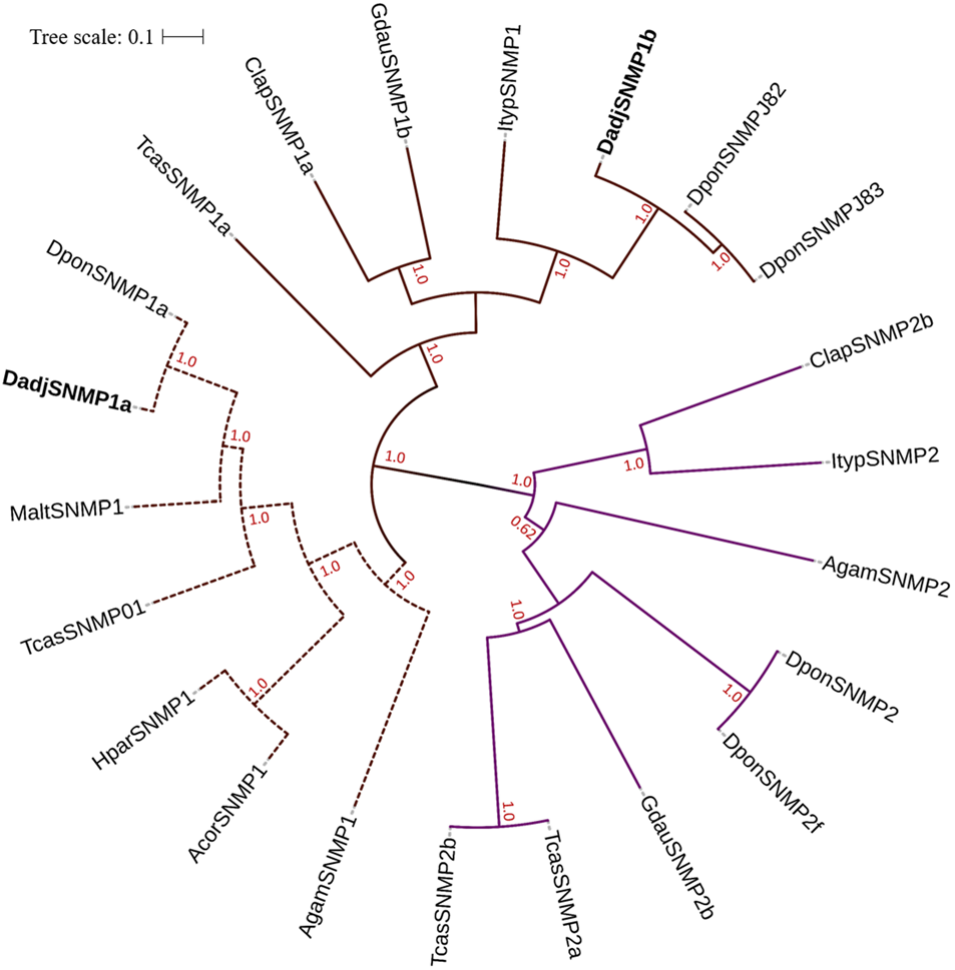
BEAST phylogenetic tree of sensory neuronal membrane proteins (SNMPs). Evolutionary Bayesian analysis of SNMPs of *Dendroctonus adjunctus* (Dadj), *D. ponderosae* (Dpon) *Ips typographus* (Ityp), *Tribolium castaneum* (Tcas), *Chrysomela lapponica* (Clap), *Galeruca daurica* (Gdau), *Anomala corpulenta* (Acor), *Holotrichia parallela* (Hpar) and *Anopheles gambiae* (Agam). Posterior probability values greater than 70% are displayed, and the tree was constructed via iTOL.

For motif analysis and phylogenetic tree reconstruction, we used a set of 24 sequences, which included DadjSNMPs and orthologs from six Coleoptera species and *Anopheles gambiae* (supplementary Table S4). We found four motifs that were present in 100% of the SNMPs, distributed in a 2-4-3-1 pattern (Fig. 7). These motifs correspond to four regions within the extracellular loop, of which the residues are conserved in more than 80% of the sequences. Motifs 2, 4, and 3 include the two domains that have relatively high sequence conservation in the ectodomain region. Finally, the phylogenetic tree was divided into two subclades, which grouped the SMP1 and SNMP2 subfamilies (Fig. 8). The two DadjSNMPs were clustered in the SNMP1 subclass related to *D. ponderosae* proteins with posterior probabilities of 100%.

## Discussion

This study represents the first analysis of the head transcriptome of *D. adjunctus* collected in infested trees during their higher incidence period and the identification of olfactory genes encoding nonreceptor proteins. Out of a total of 44,420 unigenes identified, 31.25% were annotated in the three categories of GO terms, and the most abundant functional groups showed a similar frequency in terms of molecular function, binding and catalytic activity, which is similar findings of olfactory processes and functions reported in the antennal transcriptome of other beetle species^45–51^, this indicates that it is possible to obtain similar information when including all the head, and reduces the number of individuals required to perform RNA-seq, in this study 120 heads were used, while in other works, the average number for the antenna processing are 1,500 bark beetles. On the other hand, considering that nonmodel organisms generally have limited genomic or transcriptomic datasets^52^, the low percentage of annotated genes may be due to a large number of genes that are not homologous to those with GO terms, indicating high levels of unknown processes in this tissue.

Of the total number of translated genes, 57.14% had a significant similarity with those in the UniProtKB Insecta database, and more than 50% of the transcripts were related to *D. ponderosae*, whose genome has been completely sequenced^53^. In this research, we identified thirty-six nonreceptor olfactive genes from the head transcriptome of *D. adjunctus* by homology, which is greater than that reported in the antennal transcriptome of *D. valens* (32) and *I. typographus* (24) but lower that that reported for *D. ponderosae* (45) and *T. castaneum* (73). It has been suggested that differences in the number of chemosensory genes among related insect species may be due to physiological and behavioral adaptations in specific environments that can lead to the gain or loss of functional genes^54–56^. However, the expression of members of these multigenic families has been reported in nonsensory structures. To obtain the total number of nonreceptor genes, it is necessary to explore other sensory organ tissues at different stages of development.

Insect OBPs are a multigenic family that includes different members with distinct characteristics^57^. In the transcriptome of *D. adjunctus*, a total of 27 OBPs were identified and classified into the classic (16), minus-c (10) and plus-c (1) subclasses. To perform de novo motif analysis, DadjOBPs and orthologous protein sequences were divided into the identified subgroups, which then allowed us to obtain results with higher statistical significance and biological sense. The motif patterns exhibited the main characteristics of the three subclasses of OBPs, in addition to highly conserved residues in all OBPs.

Phylogenetic analysis showed a division of the DadjOBPs and OBPs of five species of Coleoptera according to the subfamily to which they belong. Although OBPs are a highly divergent group, the tree branches include different taxa that delimit groups with high similarity in sequences and those with the same motif patterns, suggesting the occurrence of functional differences; moreover, this could be a clue for the characterization of these proteins. Most of the DadjOBPs identified exhibit characteristics of the classic subfamily, which appear to play a relatively general role in the transport of odorants and sex pheromones^58^.

In the motif pattern analysis, we found classic OBP (DadjOBP21 and DadjOBPJ75) sequences with characteristics reported in PBPs and GOBPs in Diptera and Lepidoptera, which had motifs conserved between C3–C5 and an additional motif in the N-terminal region (patterns 6C and 7C)^59–62^. PBP and GOBP groups in Lepidoptera have shown high specificity for host volatiles and pheromones, and similar proteins have been reported in Diptera, Hymenoptera, and Coleoptera. However, they have not been identified in any bark beetle transcriptome, even though semiochemicals influence the population dynamics of *Dendroctonus* species^14,63,64^. In this sense, the results of the phylogenetic reconstruction, in which OBPs with these patterns clustered with proteins from other Coleoptera species that have been classified in a specific PBP/GOBP lineage^49^, suggest the presence of this subgroup of OBPs, so they could be candidates for structural and docking studies with homology models^65–68^.

The identification of *D. adjunctus* OBPs, with characteristics of members of the minus-C and plus-C subfamilies, was supported by the results of the phylogenetic analysis. The minus-C DadjOBPs clustered in an internal clade related to the classic and plus-C OBPs. It has been suggested that this distribution shows evolutionary patterns on both short (same genus) and long-term scales (between insect species) and indicates a rapid evolutionary divergence of the three subfamilies^24^. On the other hand, the distribution of minus-C OBPs within the phylogenetic tree may coincide with the hypothesis that minus-C OBPs could be ancestral proteins, and the driving force in the evolution of OBPs is oriented to the introduction of major complexity, which is associated with the number of disulfide bridges^69,70^.

For the plus-C subclass, we identified only one protein (DadjOBP2) that had an identity greater than 90% with DponOBP2a/DponOBP2b and the same motif pattern. Although there is a high identity among closely related species, a high variability of plus-C members among insects has been reported^67^, which coincides with the low similarity (< 45%) between DadjOBP2/DponOBP2 with their homologs in *I. typographus* and the lack of two motifs in the N-terminus. However, information on the binding affinities of the minus-C and plus-C subfamilies is limited, and members of these families have been reported not only in antennae and labial and maxillary palps but also in nonsensory structures^72–76^; therefore, additional research is needed on the structure and physiological function of nonclassic OBPs.

CSPs compose a family of soluble proteins that have functions similar to those of OBPs in the recognition and transport of exogenous hydrophobic molecules^19^. We identified seven CSPs for *D. adjunctus*, and compared to OBPs, the members of this family present a smaller divergence in the sequences of the different Coleoptera species^74^. Although the CSPs presented less than 40% sequence identify, all the sequences encoded motifs that represent the four conserved cysteine profiles, and more than 50% of the CSPs have the same motif pattern. In addition, the clusters in the phylogenetic tree had similar motif patterns, indicating an origin from a speciation process, whose variation is the result of diversification of amino acid sequences^74^. DadjCSPs were dispersed among different branches of the phylogenetic tree, grouped with CSPs of *D. ponderosae* and *D. armandi*, which have been reported with specific expression patterns in antenna and mouth tissues^66^.

SNMPs are OSN membrane proteins that are associated with chemosensory neurons in insects and are classified into two subgroups: SNMP1 and SNMP2^32^. In this study, only two proteins homologous to SNMP1a and SNMP of *D. ponderosae* were identified in the transcriptome of *D. adjunctus*. The phylogenetic tree was divided into both subgroups, and the DadjSNMPs clustered in the SNMP1 subclade. This division has been reported for SNMPs of different species of insects^36,75^. Some authors have suggested that the SNMP family originated through duplication events, which contributed to the formation of both subgroups that have diverged over a long period of evolution^33^. This idea is consistent with the low similarity that was observed between the SNMP1 and SNMP2 subfamilies. However, both subgroups exhibited patterns of four conserved motifs, representing the characteristic regions of this family, and the similarity between homologous proteins within each subgroup may suggest a negative selection in their primary structure^75^.

Several studies^18,76–78^ have demonstrated the exclusive or primary expression of SNMP1 in insect antennae and support the model that this protein may be involved in the detection of pheromones and host volatiles. The identification of only two members of the SNMP1 subgroup in the head transcriptome of *D. adjunctus* collected from freshly infested trees and their homology to SNMP1 expressed in the antennae of *D. ponderosae*, *D. valens* and *I. typographus* suggest similar functions involved in bark beetle host searching behavior. Additionally, different studies have shown that SNMP2 is expressed in different parts of the body^33,36,77–79^, which supports the hypothesis that the presence of SNMP2 is not limited to the antennae and that it may be involved in different physiological processes, such as taste and tactile sensation.

The nonreceptor olfactory genes identified in the head transcriptome of *D. adjunctus* and the analysis of these genes with those of other species of Scolytinae and Coleoptera increase the amount of information on the molecular basis of the olfactory system in bark beetles. The inclusion of a comparative analysis of sequence motifs of OBPs, CSPs, and SNMPs provides clear information on the distinct characteristics of each family and their subclasses. These results support the classification of OBPs and CSPs based on the number of conserved cysteine residues in the primary sequence and could be applied as a reference for the naming and grouping of the nonreceptor genes.

The integration of motif patterns into phylogenetic trees allowing not only an improved understanding of the evolutionary process but also the conservation of motif patterns between nonreceptor protein families of different Scolytinae and Coleoptera species may suggest distinct regions with functional or structural importance. As the biological importance of a region in a protein increases in evolution, the evolutionary pressure on the region becomes higher, making it more invariable or conserved^80^. Finally, the relation of DadjCSPs and DadjSNMPs with homologs involved in host volatile and pheromone detection provides a useful resource for future research on different *Dendroctonus* species.

## Materials and methods

### Insect collection

Mature adults of *D. adjunctus* were collected manually under the bark of three recently infested pine trees (*Pinus oocarpa* Schiede ex Schltd.) in their major dispersal and colonization period, at the end of October 2018 in Military Zone 31 Rancho Nuevo, San Cristobal de las Casas, Chiapas, Mexico. To keep the RNA intact, the beetles were preserved in microtubes with 80 µl of RNAlater Storage Solution (Thermo Fisher Scientific). The tubes were then incubated overnight at − 4 °C and remained at − 20 °C until processing.

### Total RNA extraction, construction of cDNA libraries and BGI sequencing

The species of the collected bark beetles were verified with taxonomic keys^81^ and sexed according to their genitalia. The heads were removed and separated by sex, and all samples were stored at − 20 °C in RNAlater. Total RNA extraction was performed for three pools of 40 heads (20 females and 20 males) via an SV Total RNA Isolation System (Promega) according to the manufacturer's instructions, after which the quality and concentration of RNA were checked via a NanoDrop 2000 instrument. Construction of the three cDNA libraries and sequencing via the BGISEQ-500 platform were performed at BGI, Hong Kong, China.

### Cleaning and de novo assembly

The quality of the three libraries was verified with FastQC v0.10, and the cleaning of adapters and low-quality readings was performed with FastP. Assembly of the three libraries was carried out with Trinity v2.0.6, with the default value of kmer = 25, and the quality of the complete assembly was verified with rnaQUAST v.0.3.08. The total number of contigs generated with Trinity, the N50 length, average length, and percentage of GC were recorded, and the redundancies were eliminated to obtain the final number of unigenes.

### Gene ontology (GO) and homology analysis

The mapping routine of HMMER2GO v0.17.9 was used against a customized HMM Pfam database to obtain information on the molecular functions, cellular components, and biological processes associated with the unigenes, and the results were visualized in WEGO 2.0. Homology analysis was performed with BLASTx against a dataset that was constructed from the Insecta UniProtKB database, with an E-value of 1e−6. The results were imported into Blast2GO to obtain the distribution of the species, the E-value and the percentage of similarity found among the hits, and unigenes whose description corresponded to the genes of nonreceptor proteins involved in the reception of odors were filtered and removed.

### Classification of nonreceptor proteins and functional analysis

The longest ORF and the probable coding regions for OBPs, CSPs, and SNMPs were predicted with Transdecoder (https://github.com/TransDecoder/TransDecoder), with a minimum length of 100, and as a criterion for the retention of reading frames, a homology test was included in order to maximize sensitivity and obtain functionally significant ORFs. Predicted ORFs for nonreceptor proteins were used for functional annotation from domain searches in the PFAM, SUPERFAMILY, CATH-Gene3D, and PANTHER databases.

The predicted ORFs for OBPs, CSPs and SNMPs were used for pattern analysis and functional motif discovery. The sequences of the OBP and CSP families of *D. adjunctus* were compared with the orthologous sequences of four species of Scolytinae and the model beetle *T. castaneum*, while *D. adjunctus* SNMPs were grouped with their homologous proteins of *D. ponderosae*, *I. typographus*, *T. castaneum*, *Chrysomela lapponica* Linnaeus, *Galeruca daurica* Joannis, *Anomala corpulenta* Motschulsky, *Holotrichia parallela* Motschulsky and *Anoplophora gambiae* Giles*.* The protein classification was performed based on CLUSTAL O alignments, with the default parameters of penalty for gaps, and under the conserved cysteine profiles.

Sequence motif analysis was performed via MEME 3.5.78^82^. The parameters assigned for the OBPs and CSPs were as follows: minimum width = 6, maximum = 10, the maximum number of motifs to be found = 8. For SNMPs, there parameters were as follows: minimum = 40 maximum = 95 and number of motifs = 10. In all three cases, motifs with p < 0.0001 were selected. Furthermore, the candidate OBPs and CSPs were searched for the presence of signal peptides using SignalP 4.0 (https://www.cbs.dtu.dk/services/SignalP/), and the transmembrane domains of the candidate SNMPs were predicted using TMHMM v3.0 (https://www.cbs.dtu.dk/services/TMHMM/).

### Phylogenetic analysis

For the phylogenetic tree reconstruction, we used the sets of the predicted protein sequences of DadjOBPs, DadjCSPs and DadjSNMPs together with orthologs of Scolytinae and Coleoptera species that were independently analyzed (supplementary Table S4). The sequences were aligned using the CLUSTAL O algorithm, and the best protein evolutionary model was searched in ModelTest-NG with the AIC and BIC. The phylogenetic tree was reconstructed with Bayesian inference using the Markov chain Monte Carlo method by the program BEAST v1.10.4 in conjunction with a WAG^83^ starter model and 4,000,000 generations, and the diagnosis of the MCM output was observed in Tracer. For the annotation, 30% of the trees were generated, initial probabilities were discarded, and the subsequent probability was determined for the remaining trees. The consensus trees were visualized and edited in iTOL. Last, the sequences of *D. adjunctus* were submitted to the NCBI database to obtain the access numbers for DadjOBPs (MT604218-MT604241), DadjCSPs (MT520150-MT520156) and DadjSNMPs (MT604216 y MT604217). Vouchers specimens CEAM-0051 “Colección Entomológica del Colegio de Postgraduados”.

## Supplementary information

Supplementary Information.
