# Supplementary material for: Identification and motif analyses of candidate nonreceptor olfactory genes of Dendroctonus adjunctus Blandford (Coleoptera: Curculionidae) from the head transcriptome
Source: Sci Rep. 2020 Nov 26;10:20695. doi: 10.1038/s41598-020-77144-5 (PMC7691339; doi:10.1038/s41598-020-77144-5)
Supplement: Supplementary file 1 — Supplementary Information. [file 41598_2020_77144_MOESM1_ESM.pdf]

## Supplementary Information for

# Identification and motif analyses of candidate nonreceptor olfactory genes of *Dendroctonus adjunctus* Blandford (Coleoptera: Curculionidae) from the head transcriptome

Brenda Torres Huerta[1], Obdulia L. Segura-León[1]*, Marco A. Aragón-Magadan[1] & Héctor González-Hernández[1]

[1]Entomology and Acarology Program, Colegio de Postgraduados Campus Montecillo, Mexico-Texcoco, km. 36.5, C. P. 56230, Montecillo, State of Mexico, Mexico. *Corresponding author

**E-mail addresses:**

Brenda Torres Huerta: brendth@gmail.com

Obdulia Lourdes Segura León: sleon@colpos.mx

Marco Aurelio Magadan Aragón: marco.magadan@gmail.com

Héctor González Hernández: hgzzhdz@colpos.mx

**supplementary Table S.1.** Overview of the sequencing and assembly process

| Samples | Raw reads | Mean length (bp) | Clean data | | | |
|---|---|---|---|---|---|---|
| | | | Read number | GC (%) | Reads Q20 | Reads Q30 |
| 1 | 95,865,730 | 100 | 95,865,730 | 38.41% | 98.39% | 92.67% |
| 2 | 96,397,504 | 100 | 96,397,504 | 37.25% | 98.39% | 92.65% |
| 3 | 97,612,326 | 100 | 97,612,326 | 35.01% | 98.32% | 92.35% |

**Combined Trinity assembly of the three samples**

| | |
|---|---|
| Total number | 71,174 |
| Mean length (bp) | 574 |
| Unigen N50 | 3,126 |
| GC (%) | 41.21% |

Each sample contains a mixture of sixty heads of males and females of *D. adjunctus*.

**supplementary Table S2.** BLASTx annotation against the UniprotKB database for putative nonreceptor protein of *D. adjunctus*

| Gene name | Gene ID | ORF (bp) | SP | ORF C | Best BLASTX match | | | | |
|---|---|---|---|---|---|---|---|---|---|
| | | | | | Name | Acc No. | Specie | Evalue | Identity (%) |
| DadjOBP2 | DN2771g2 | 267 | 1-21 | Y | **Odorant-binding protein** 2 | M4VUV4 | *Dendroctonus ponderosae* | 3.78E-33 | 93.99 |
| DadjOBP3 | DN5192g2 | 123 | 1-18 | N | Odorant-binding protein 3 | M4VR73 | *Dendroctonus ponderosae* | 5.04E-42 | 84.416 |
| DadjOBP4 | DN81g1 | 414 | 1-18 | Y | Odorant-binding protein 4 | N6UHS9 | *Dendroctonus ponderosae* | 2.0E-102 | 95.276 |
| DadjOBP5 | DN6336g2 | 389 | 1-25 | Y | Odorant-binding protein 5 | M3UZD2 | *Ips typographus* | 8.16E-40 | 50.932 |
| DadjOBP6 | DN510g1 | 141 | 1-23 | N | Odorant-binding protein 6 | M4VTI4 | *Dendroctonus ponderosae* | 1.39E-85 | 94.118 |
| DadjOBP8 | DN521g1 | 128 | 1-15 | N | Odorant-binding protein 8 | M4VRL6 | *Dendroctonus ponderosae* | 6.61E-75 | 90.598 |
| DadjOBP9 | DN2638g1 | 112 | 1-19 | N | Odorant-binding protein 9 | M4VRM7 | *Dendroctonus ponderosae* | 2.59E-45 | 89.873 |
| DadjOBP11 | DN2650g1 | 117 | 1-16 | Y | Odorant-binding protein 11 | M4W9C1 | *Dendroctonus ponderosae* | 6.11E-71 | 91.379 |
| DadjOBP11t | DN3950g1 | 127 | 1-22 | Y | Odorant-binding protein 11 | D6WZK0 | *Tribolium castaneum* | 8.32E-48 | 64.486 |
| DadjOBP12 | DN3177g1 | 170 | 1-22 | Y | Odorant-binding protein 12 | M3VHC4 | *Ips typographus* | 3.27E-82 | 72.316 |
| DadjOBP12l | DN2429g1 | 147 | 1-15 | Y | Odorant-binding protein 12 | A0A0B4KZC3 | *Lissorhoptrus oryzophilus* | 8.24E-36 | 43.796 |
| DadjOBP13a | DN4937g1 | 156 | 1-44 | N | Odorant-binding protein 13 | M4VRL1 | *Dendroctonus ponderosae* | 5.32E-81 | 86.26 |

| Gene name | Gene ID | ORF (bp) | SP | ORF C | Best BLASTX match | | | | |
|---|---|---|---|---|---|---|---|---|---|
| | | | | | Name | Acc No. | Specie | Evalue | Identity (%) |
| DadjOBP13b | DN81g1 | 149 | 1-19 | Y | Odorant-binding protein 13 | J3JVJ5 | *Dendroctonus ponderosae* | 4.16E-52 | 97.973 |
| DadjOBP15 | DN16961g1 | 213 | NO | N | Odorant-binding protein 9 | A0A0S3J3B4 | *Colaphellus bowringi* | 2.33E-26 | 38.281 |
| DadjOBP16 | DN1281g2 | 118 | 1-17 | N | Odorant-binding protein 16 | M4W9C3 | *Dendroctonus ponderosae* | 4.79E-70 | 85.47 |
| DadjOBP18 | DN3129g2 | 142 | 1-18 | Y | Odorant-binding protein 18 | A0A0H3W579 | *Dendroctonus ponderosae* | 7.12E-80 | 87.234 |
| DadjOBP21 | DN5264g1 | 136 | 1-19 | Y | Odorant-binding protein 21 | A0A0H3W5P0 | *Dendroctonus ponderosae* | 2.96E-93 | 96.296 |
| DadjOBP22 | DN4747g1 | 149 | 1-19 | Y | Odorant-binding protein 22 | M4VRM1 | *Dendroctonus ponderosae* | 1.16E-84 | 89.552 |
| DadjOBP23 | DN5554g1 | 140 | 1-17 | Y | Odorant-binding protein 23 | A0A2P9JZF8 | *Anthonomus grandis* | 2.06E-44 | 46.715 |
| DadjOBP24 | DN3480g1 | 132 | 1-15 | N | Odorant-binding protein 3 | A0A1C8K2C2 | *Rhynchophorus ferrugineus* | 3.22E-17 | 34.375 |
| DadjOBP27 | DN13064g1 | 131 | 1-21 | N | Odorant-binding protein 27 | M4VTJ4 | *Dendroctonus ponderosae* | 4.11E-78 | 87.402 |
| DadjOBP30 | DN81g1 | 134 | 1-18 | Y | Odorant-binding protein 30 | M4W9B7 | *Dendroctonus ponderosae* | 1.36E-90 | 97.744 |
| DadjOBP31 | DN3950g1 | 127 | 1-22 | Y | Odorant-binding protein 11 | D6WZK0 | *Tribolium castaneum* | 8.32E-48 | 64.486 |
| DadjOBPJ74 | DN3724g1 | 124 | 1-18 | N | Odorant-binding protein | I1VJ74 | *Dendroctonus ponderosae* | 9.83E-75 | 93.86 |
| DadjOBPJ75 | DN1254g1 | 136 | 1-19 | Y | Odorant-binding protein | I1VJ75 | *Dendroctonus ponderosae* | 6.27E-96 | 97.778 |
| DadjOBPJ79 | DN1223g1 | 146 | 1-21 | Y | Odorant-binding protein | I1VJ79 | *Dendroctonus ponderosae* | 1.29E-98 | 92.414 |

| Gene name | Gene ID | ORF (bp) | SP | ORF C | Best BLASTX match | | | | |
|---|---|---|---|---|---|---|---|---|---|
| | | | | | Name | Acc No. | Specie | Evalue | Identity (%) |
| DadjOBPJ77 | DN3724g1 | 138 | 1-19 | N | Odorant-binding protein | I1VJ77 | *Dendroctonus ponderosae* | 2.37E-93 | 94.161 |
| DadjCSP1 | DN936g1 | 121 | 1-16 | Y | **Chemosensory protein** 1 | A0A0H3W576 | *Dendroctonus ponderosae* | 7.90e-44 | 80.381 |
| DadjCSP2 | DN8588g1 | 122 | 1-18 | Y | Chemosensory protein 2 | M4VTH9 | *Dendroctonus ponderosae* | 3.62e-78 | 90.909 |
| DadjCSP3 | DN4032g1 | 138 | 1-19 | Y | Chemosensory protein | I1VJ19 | *Dendroctonus ponderosae* | 7.85E-91 | 91.971 |
| DadjCSP4 | DN665g1 | 296 | NO | N | Chemosensory protein 4 | A0A0H3W5L5 | *Dendroctonus ponderosae* | 2.6e-146 | 81.343 |
| DadjCSP6 | DN20762g1 | 154 | NO | N | Chemosensory protein 6 | M4VTH1 | *Dendroctonus ponderosae* | 9.11E-88 | 91.538 |
| DadjCSP8 | DN18155g2 | 150 | NO | Y | Chemosensory protein 8 | M4VR62 | *Dendroctonus ponderosae* | 6.80e-86 | 95.276 |
| DadjCSP9 | DN204g2 | 116 | 1-25 | Y | Chemosensory protein 9 | A0A345BT19 | *Dendroctonus ponderosae* | 4.20E-68 | 94.783 |
| DadjSNMP1 | DN1661g1 | 520 | - | Y | **Sensory neuron membrane protein** 1a | M4W9B3 | *Dendroctonus ponderosae* | 0.0E+00 | 85.336 |
| DadjSNMP | DN2852g1 | 543 | - | Y | Sensory neuron membrane protein 1b | I1VJ82 | *Dendroctonus ponderosae* | 0.0E+00 | 94.702 |

ORF: Open reading frame, SP: Signal peptide, ORF-C: Open reading frame complete, Acc. No: Accession number

**supplementary Table S3.** Functional annotation of the DadjOBPs and DadjCSPs by searching for domains in the PFAM, SUPERFAMILY and CATH-Gene3D databases

| | **IPR006170** | | **IPR036728** | | | |
|---|---|---|---|---|---|---|
| | **PFAM (PF01395)** | | **SUPERFAMILY (SSF47565)** | | **CATH-Gene 3D (1.10.2080.10)** | |
| OBP | Range | E-value | Range | E-value | Range | E-value |
| DadjOBP2 | N | N | 123-219 | 8.72E-7 | N | N |
| DadjOBP4 | 18-127 | 2.0E-19 | 21-127 | 1.12E-21 | 21-128 | 7.7E-21 |
| DadjOBP5 | N | N | 297-383 | 1.83E-7 | 288-385 | 2.4E-8 |
| DadjOBP6 | 28-131 | 1.6E-15 | 27-139 | 5.76E-22 | 24-140 | 5.8E-23 |
| DadjOBP8 | 18-123 | 5.8E-16 | 26-113 | 7.85E-18 | 10-121 | 1.4E-18 |
| DadjOBP11 | 32-100 | 6.3E-6 | 24-100 | 4.58E-10 | 2-107 | 6.8E-10 |
| DadjOBP12 | 35-164 | 5.6E-9 | 80-165 | 3.14E-10 | 29-172 | 1.6E-10 |
| DadjOBPL12 | 20-134 | 1.7E-14 | 26-140 | 4.19E-21 | 18-144 | 6.0E-23 |
| DadjOBP13I | 43-149 | 2.9E-8 | 46-150 | 8.37E-13 | 42-155 | 2.8E-12 |
| DadjOBP13II | 18-127 | 2.0E-19 | 21-127 | 1.15E-21 | 21-128 | 7.7E-21 |
| DadjOBP15 | 97-201 | 3.8E-11 | 79-208 | 1.96E-14 | 86-202 | 8.4E-18 |

| | | | | | | |
|---|---|---|---|---|---|---|
| DadjOBP16 | 24-101 | 2.1E-7 | 21-110 | 1.31E-11 | 19-117 | 1.6E-9 |
| DadjOBP18 | 34-138 | 1.9E-16 | 35-138 | 2.88E-22 | 33-139 | 2.7E-20 |
| DadjOBP21 | 23-129 | 2.4E-8 | 28-129 | 4.58E-14 | 23-130 | 1.2E-12 |
| DadjOBP22 | 23-129 | 2.4E-8 | 28-129 | 4.58E-14 | 23-130 | 1.2E-12 |
| DadjOBP23 | 20-126 | 2.8E-18 | 25-127 | 3.01E-25 | 22-127 | 3.9E-22 |
| DadjOBP24 | 21-125 | 1.9E-15 | 23-127 | 8.63E-20 | 24-127 | 3.6E-19 |
| DadjOBP27 | N | N | 26-118 | 1.01E-5 | 23-122 | 1.5E-8 |
| DadjOBP30 | 18-127 | 2.0E-19 | 21-127 | 1.15E-21 | 21-128 | 7.7E-21 |
| DadjOBPJ74 | 19-109 | 2.0E-12 | 19-112 | 4.97E-15 | 18-117 | 4.5E-17 |
| DadjOBPJ75 | 22-127 | 8.0E-22 | 21-134 | 5.49E-31 | 17-134 | 5.4E-31 |
| DadjOBPJ77 | 25-128 | 1.5E-15 | 24-136 | 5.36E-22 | 22-137 | 3.8E-23 |
| DadjOBPJ79 | 26-135 | 1.5E-16 | 28-144 | 1.07E-18 | 25-145 | 2.1E-22 |
| **CSP** | Rango | E-value | Rango | Evalue | Rango | E-value |
| DadjCSP1 | 19-110 | 1.9E-37 | 19-122 | 1.7E-41 | 16-124 | 5.3E-44 |
| DadjCSP2 | 21-111 | 6.1E-35 | 21-120 | 1.96E-38 | 19-121 | 7.4E-39 |

| | | | | | | |
|---|---|---|---|---|---|---|
| DadjCSP3 | 27-118 | 1.8E-36 | 27-126 | 6.15E-40 | 24-130 | 3.5E-46 |
| DadjCSP4 | 56-146 | 5.2E-29 | 56-156 | 6.67E-34 | 53-158 | 1.6E-33 |
| DadjCSP6 | 48-138 | 2.3E-34 | 48-151 | 1.31E-38 | 45-153 | 1.7E-41 |
| DadjCSP8 | 44-136 | 2.5E-37 | 44-148 | 4.18E-40 | 42-149 | 3.7E-44 |
| DadjCSP9 | 38-113 | 1.3E-15 | 37-113 | 3.79E-22 | 34-115 | 6.5E-19 |

OBPs: PFAM (PF01395): familia PBP/GOBP; SUPERFAMILY (SSF47565) y CATH-Gene 3D (G3DSA:1.10.238.20): insect pheromones/odor binding proteins; CSPs: PFAM (PF03392) y CATH-Gene 3D (1.10.2080.10): Insect Odour Binding Protein A10/ Ejaculatory bulb-specific protein 3; SUPERFAMILY (SSF100910): Chemosensory proteins.

**supplementary Table S4. supplementary Table S3.** Functional annotation of the DadjSNMPs by searching for domains in the PFAM and PANTHER databases

| | IPR002159 | | | |
|---|---|---|---|---|
| | **PFAM (PF01130)** | | **PANTHER (PTHR11923)** | |
| SNMP | Range | E-value | Range | E-value |
| DadjSNMP1a | 11-472 | 1.6E-116 | 1-507 | 1.0E-191 |
| DadjSNMP1b | 18-482 | 9.3E-119 | 12-498 | 7.5E-191 |

PFAM (PF01130): CD36 family**;** PANTHER (PTHR11923): Scavenger receptor Class B type-1 SR-B1

```
                       *        20         *        40         *        60         *        80         *       100         *       120
DadjOBP6   : --------------------------------------------MF----KSL-SVLL---LIL-VVGSLDAKITLPPELQE-YVDDLHKLCLEKG---GLT--ENDHQTYDI--------------- :  54
DadjOBP11T : --------------------------------------------MKPIIGARD-FLLSVAVILL-ICGKKVKCFTSED--VA-NDLKFIKICKSNSPPGAYS--MNDVLDTKN-------------AE :  64
DadjOBP12  : -----------------------------------------------MFGFRKNVLVLVLVVFESLALQKNNKCDIPLSAPK-RIEEVINTCQDEI---KIAILSEALEAFKVNEHQVVSRAKRSAFN :  77
DadjOBPL12 : ----------------------------------------------------MNILVFICVLVGVKALDQT----LVELMQA-KIQEFGISCSEQE---NAS--EDDIAALL--------E-----KR :  53
DadjOBP15  : MGGGHYSVPAIIYPLTAGMRWKSAFRAILALFRSGSGKIMRIMSLFQPVDVRMKWARFALFFVSIVAVAHG-----SSKENP-RLEAWLRNCQNET---GAS--EDDFQIIK--------S-----RK : 104
DadjOBP21  : --------------------------------------------MA----LTT-WVLSIMLIL----P---AARALSDEMKE-LVQMLHNTCVAET---GVN--EDLIQKVNAE------------KI :  54
DadjOBP27  : --------------------------------------------MH--------FLFIVALTLY-L--------PISNG------FLTVPKCLIST---GAR--IKDLDNLAT--------------G :  42
DadjOBP30  : --------------------------------------------MR----NL--LKLSIAFAV----VSVISCQDFTEEQRK-KIIENRQQCIEET---KVN--PDLIEKADL-------------GD :  55
DadjOBP31  : --------------------------------------------MYF-----Q-WGVSVLLCIL--GVAQLVAAGKPDGLFARMTPADLEMCGKDT---GVD--RKEFEEARE--------------K :  57
DadjOBPJ75 : --------------------------------------------MK----TAL-KVFLLALAI----P---TIMGMSDELQE-LANQLHTTCIGET---GAA--EDAITNARN-------------GD :  53
DadjOBPJ77 : --------------------------------------------------RCL-RVCLIVFVSL-CGFSCSLKITLPPELQE-YVDDLHKLCLEKG---GLT--ENDHQTYDI--------------- :  55
DadjOBPJ79 : --------------------------------------------ML----TK--TILIWAAILLTVFVSKGNCRLTEKQLAA-AVKLVRNMCMGKS---KVN--PEDIDKMHQ-------------GN :  59
DadjOBP16  : --------------------------------------------------MGPTILLFVALVIMTNAY--------VPNVND-K---IRNFCIDDS---GVS--VEMVENLLA-------N-----PE :  49
DadjOBP8   : ----------------------------------------------------MKLLIFASILVCASAQDQA----WRDNMKE-KLTEFGIECAESE---QAT--PEDIEALH--------N-----HK :  53
DadjOBPJ74 : ------------------------------------------------------MKLMWILVL-GAALKIADGAMTEAQMKA-ALKLIRNVCQPKN---KVT--NEQIAAMHN-------------GD :  54
                                                                                                       C1

              *       140         *       160         *       180         *       200         *       220         *       240
DadjOBP6   : NDKNEKMMCYMKCLMLESKWMK-SGGEIDY----DFIE-TQ--AYPEVKDLLLNALSKCRTI-----------EEG--ADLCEKSYNFNKCMYEADPVNWF-----FV------------ : 136
DadjOBP11T : NTHSRSFKCFLHCLLTKYGWMDEDGGYLLH----DIRE-TL--QQSDLQLATLEYILYTCTA-----------VKS--SDRCQRAHFFTDCFWKKMDEEQPTADELFYNVKTRK------ : 158
DadjOBP12  : EDEKKIAGCLLQCVYRKLNAVNE-FGFPTVEGLVSLYTEGV--TQKEYIAATRQAVTKCLDNAQNAHEIGAKPVEA--SKSCEVAYEVFDCVSLEVAKYCG-QTP--------------- : 176
DadjOBPL12 : MPSSHEGKCVIFCGAKKLNIMHE-DGSFGE-GYVEWLNKAK-ADDPDFYNKLMEIKGKCEAE--------VEHLSL--SDSCEKAASLADCSQQESRKNGL-DKFLF------------- : 146
DadjOBP15  : IPTSKEGVCMVECLFTKLHIID--NGQFNQRGFVITFSPIA-RGNLKKLAALKEVAALCRAE--------VSAVQT--GEQCSATRTVLDCFGKNMDKLAI-AKKP-------------- : 196
DadjOBP21  : FADDENLKCYIKCLMAQMACID-DDGIIDE----EATI-AI--LPEEYQALAAPVIRACGT------------KHG--ANPCENAWLSHRCYAEMEPSAYI-----LI------------ : 135
DadjOBP27  : NSLPESSRCFVKCVGEESGLIRN--GILHS----EHFDAFP--MVSRLKGDVLVDVRRCMES-----------VQGIKIESCKDVDNLNDCMKIAYRQKYSDPK---------------- : 127
DadjOBP30  : FAEDQALKCFTKCFYQKAGFVN-DKGEVQK----DVVE-AK--LPPQADKKRALEIVDKCA------------LKG--KDACETVYLIHKCYFEHTHPEAD-----EKSAKDGKSDEKKA : 148
DadjOBP31  : RALNHSMLCFLKCAMEKVGFLND--GHLEV----DQAK-ES--FPDRM----TEPLVECFKA-----------VGP--ISTCDDVQKVEDCLPSS------------------------- : 126
DadjOBPJ75 : FSEADSFKCYIKCLLSQMAIIDDDDGTIDV----DAMV-AV--LPEEIQEATEPIIRKCGS------------IIG--ANPCDSAWLTHKCYYKEGPEHYF-----LI------------ : 135
DadjOBPJ77 : NDKNEKMMCYMKCLMLESKWMK-SGGEIDY----DFIE-TQ--AYPEVKDLLLNALSKCRTI-----------EEG--ADLCEKSYNFNKCMYEADPVNWF-----FV------------ : 137
DadjOBPJ79 : WDVAYEAQCYMWCGFNMYKMLDK-ENHFDKPNALKQMD--Q--LPIDLQDYAIKCMDQCENA-----------VTN-FDDKCVVAFEYSKCLYFCDPEKYFLP------------------ : 145
DadjOBP16  : KDLIDVESCYVHCIFTEMGLLSE-NGNVEIENFESLKASEAPYIDLNCLDKIKSIDNCCEMM-------------L--LRACHV------------------------------------ : 117
DadjOBP8   : PPVTHAGKCVIFCVSKKLNLMNA-DGTLNVTPQTDWIEKVK-ETDSEAFGKMKTIYHHCADT--------GRTETL--FSIHSSTK---------------------------------- : 127
DadjOBPJ74 : WNQDKNGMCYMNCVLNYYKLQLP-DNSFDWETGLKVVE-SQ--APPSMVGFIVETITGCKDA-----------GKI-IS---------------LSPPNW-------------------- : 123
                     C2  C3                                            C4                      C5       C6
```

**supplementary Figure S1.** Multiple sequence alignment of *D. adjunctus* classics OBPs, amino acid sequences were aligned by Clustal Omega and edited using Genodoc. Green boxes show conserved cysteine residues.

```
                      *        20         *        40         *        60         *        80         *       100         *       120
DadjOBP4   : ----MQFLFAAVLVIA--------LAQVNSLTDKQKELLTQHYNQCVA--ISKVDQAVLQKARAG-DFANDANLKTHIKCISEKIGFQGTDGKFRRDVIEKKLKETIPGDN-AKNAKLIETCVVANK : 111
DadjOBP11  : MQSLVVFCALLVLALAH-----------------DPHGLESVHKKCH-----------------------NEVDSQHYLCMAKGLDLVTPEGKVNVNGVKTHVGHVESES--AKIDQIAKECAVDHA :  85
DadjOBP13a : ------MFPAMKLLLV--------LGLGAVVTRADRQQVVDFHRPCLD--HHGVEDDDLHFALDKLKIRDDDEFYLHFFCVAKQGKLMTEDGTVETDDFETKMKGIIDEDNMENVAAIVRMCLIQRD : 111
DadjOBP18  : MNGLPVFFLLLLAAVVKSDFDFSNYKEFEHLPGDQREKAIKIFNDCMA--ETGATHEMMEKSVAG-DIPDDIVFKNHLVCIGKKSGFIDENGMHVKEKMKEKLLLLLGDE--QMVDKILDNCFIEKG : 122
DadjOBP22  : MQQ--KFNFALGAVFV--------TFLVNVIEADQRGKIVEFQRGCME--AHGLLEDELHEIIDGKPIQ-NEAFYFHFFCVVKKAKLISDNGIVDTDHFEENLKGVIDEENMAHVAALARKCLIQRD : 114
DadjOBP23  : ---MMRCLFLVAIILV--------AVRAQILSQSEKEKMRAIHEGCMK--ETAADPALIGKAVKG-EYVDDPKLKNQIFCVTQKIGFTDESGEIMKEQTVKKLTEKFKNE--KVINAAVEKCVDKKA : 111
DadjOBP24  : -------------MVA--------RCQFSLLPED-IGKLLQTNAKCQK--KSGATQQEIMGTLRG-TFSDSPELKKHLFCLGVKLDFISKDGVFQKELIRDKMAVIVPDE--SQLEDLLEKCLMETE : 100
DadjOBP13b : MRSFAVCCVLVA--VL--------QVRGAPLTNDQKAKLDAYQQDCIS--ESKVNQLLVEQSRKG-IFSDDPAFKNFLFCFSQKAGFQDANGLIQKDVFERKVKIVVDDE--ALVAKLLEQCVAQKA : 112
DadjOBP9   : MKVLVVLCVVLIAFTVIA-----AAKKNKSNDEEKPKSYKKVFKECQKKDETRVDASIIRKLRKHKQVDLPANFGEHKLCVFPANFGEHKLCVLFFFFFFFFFFFFF------FYRHRLAEC----- : 111
                                                          C1                                C2                                        C3

               *       140         *       160         *       180         *       200         *       220         *       240         *
DadjOBP4   : DPKLQAFNAFKCLYTNAKINLL--------------------------------------------------------------------------------------------------------- : 133
DadjOBP11  : STDETVSHLFKCLEEKHVLSLA-GHVAPQHHH----------------------------------------------------------------------------------------------- : 116
DadjOBP13a : TVLETIRNAVDCFLGKDHKL----------------------------------------------------------------------------------------------------------- : 131
DadjOBP18  : SPQDTAFELAKCCHKQYHN------------------------------------------------------------------------------------------------------------ : 141
DadjOBP22  : DIFTTIKMAIDCFYTSEHKL----------------------------------------------------------------------------------------------------------- : 134
DadjOBP23  : TPSDTAFEFVKCLHAYAPEGLDIMALMA--------------------------------------------------------------------------------------------------- : 139
DadjOBP24  : NAADSVYQSIRCFAKQNLLPQ---------------------------------------------------------------------------------------------------------- : 121
DadjOBP13b : TPQDTSYYLAKCLREVVPNMEL-FKVAPEVVYVSNEKSRQALLQHKECAAQDPLGQEAIEAARKQGILFEDAKFKAYIFCFSKKSGIQRDDGSIDKDRFYKKFGEVIDKPAVVDQLGQKCLLEQDTP : 238
DadjOBP9   : ------------------------------------------------------------------------------------------------------------------------------- :   -
                        C4
```

**supplementary Figure S2.** Multiple sequence alignment of *D. adjunctus* Minus-C OBPs, amino acid sequences were aligned by Clustal Omega and edited using Genodoc. Orange boxes show conserved cysteine residues.

```
                     *        20         *        40         *        60         *        80         *       100         *       120
DadjOBP2  : MNKLVTLCAVLLGAACHLVQTYDFQDATFNEILSSDFEDIFDSLEDTYLHLRAKRNDEAVNSDEKCRRRHHRKPKLCCVEEVLDSLQEKEKEIIKLCFKDITGSVKESKPDRGFGNHRNFDLFSCESVE : 129
DponOBP2a : MNKLVTLYAVLLGAACHLVQTYDFQDATFNEILSSDFEDIFDSLDNTYLHPRAKRNEEAVNSDEKCRRRHHRKPKLCCGEDVLDALQEKEKEIVRLCFKDITGGVKESKPDRGFGNHRNFDLFSCEAVE : 129
DponOBP2b : MNKLVTLYAVLLGAACHLVQTYDFQDATFNEILSSDFEDIFDTLDNTYLHPRAKRNEEAVNSDEKCRRRHHRKPKLCCGEDVLDSLQEKEKEIVRLCFKDITGGVKESKPDRGFGNHRNFDLFSCEAVE : 129
ItypOBP2  : MNSVAVFAVLALGAVC-VIDAYNFQDEDFXSAVVVRDGRIVDSIDSGPVHPRVRRDQEAATVAEEKCPKRHRRPKLCCAEETLDALHAKKKEITKACFKEVTGLEKQDRHDHGP-HFKRFDLFNCKEVE : 127
            MN 6     6 LGA C 66  Y1FQD  F   6      I D36    6H R 4R1 EA    E    4 HR4PKLCC E  LD L  K KEI 4 CFK 6TG  K2 4 D G    4 FDLF C  VE

            *       140         *       160         *       180         *       200         *       220         *       240         *       2
DadjOBP2  : KRKSDMICVEQCKLQKQGLVSDDGSPKPEQISTYLKEAFTTQTWFEKV-SEGIVDKCVNEAINATKNPVKFYTEGNKVCSRSGIVLKHCLFNSIQLSCPADQIKDKNACERFQERAKKGKDLFDQPPGP : 257
DponOBP2a : KRKSDMICVEQCKLQKQGLVSDDGSPKPEQISTYLKEAFTAQTWFEKV-SQGIVEKCVNEAINATKNPVKFYTEGNKLCSRSGIVLKHCLFNSIQLSCPAGQIKDKNACERFQERAKKGKDLFDQPPGP : 257
DponOBP2b : KRKSDMICVEQCKLQKQGLVSDDGSPKPEQISTYLKEAFTTQTWFEKV-SQGIVEKCVNEAINATKNPVKFYTEGNKLCSRSGIVLKHCLFNSIQLSCPAGQIKDKNACERFQERAKKGKDLFDQPPGP : 257
ItypOBP2  : KRKSDMICIDQCVGQKKGLLDDSGAPIRDQLIQHLKQHFSNESWFDQTVVEKITSNCLAAAKNATETPIKFSTEGLKACNPSGITLKHCLFREIQLSCPADQIKDKTACDRFQDRIQKEIEIDDLRLAP : 256
            KRKSDMIC6 QC  QK GL6 D G P   Q6   LK2 F3 23WF     2 I   C6  A NAT  P6KF TEG K C  SGI LKHCLF  IQLSCPA QIKDK AC RFQ R  K   6 D    P

             60
DadjOBP2  : PPFDDNREE-- : 266
DponOBP2a : PPFDDNREEQI : 268
DponOBP2b : PPFDDNREEQI : 268
ItypOBP2  : DDQQ------- : 260
```

**supplementary Figure S3.** Multiple sequence alignment of *D. adjunctus* Plus-C OBPs with those of other Scolytinae species, amino acid sequences were aligned by Clustal Omega and edited using Genodoc. Pink boxes show conserved residues in 100%.

```
                    *        20         *        40         *        60         *        80         *       100         *       120         *
DadjCSP1 : ----------------------MKV-VLLLVVVVG---VAFGEEYTSKFDNVDLDQILSSDRLLRNYMNCLLDKGKCTPDGIELKKNLPDALANECSKCTPKQRDGAKKVIRYLIEHKRDYWNEVAAKYD : 104
DadjCSP2 : --------------------MKFCGVLVLL-L---QIAVCLCQTYTSRFDTINIDEILSNKRVLNNYVRCVLDEGPCTAEGRELRTHIPEALRTNCAKCTPSQQKFVRKGANFLIKNDPNQWSRITKKFD : 106
DadjCSP3 : -----------------MWKLLLAGSLLIC-IGQTPAEVTENSQYTTKYDNVDINEVLHSERLLKNYVNCLLDRGPCSPDGLELKKNMPDAIKTDCSKCSDKQREGSEAMMRFLIDNKPEYWNPLQEKYD : 112
DadjCSP4 : --------------------MLLIVVVLIGMALDLTGAKPAAKYYASKYDHIDVGAILNNRRMVNYYSACLLSQGACPPEGVELKRILPEALQTNCARCSEKQATTALMAIKRLKKEYPKIWNELSEKWD : 110
DadjCSP6 : --------------MKP------VVFLVVLASFYGLSSCTPQEKYTTKYDNIDLDSIIRNDRLLRNYIDCVLGKKKCTKDGQELKVHLPDALQSDCSKCSEAQRNGSRKIITHLLKNKRGWFNELQAKYD : 110
DadjCSP8 : MRSFRRNLRLKVNPILPVYSLILQIVILVCCAFIGLV-LADTPKYTTKYDNVDLEEIIKSDRLMKNYVNCLLEKGKCTPDGAELKRVLPDALHTECSKCSDNQKKGSRKIMRHLIDNKPEWWTELESKYD : 129
DadjCSP9 : -------------MNSHCFQLQLIALLVLVAS-V--SFVRSDATERPPISDEALEKTLSDKRYLQRQLKCAVGEAPCDPVGRRLKSLAPLVLRGSCPQCTEQEKKQIKKVLAYVQVNFPKEWNKMLQTYA : 114
                                     666               y    d   6   6   R 6  y  C1     C2  G eL4   P a6   C3 C4  2          6       5  6  k5d

                  140         *       160         *       180         *       200         *       220         *       240         *       260
DadjCSP1 : PEGAYYKKYQEQAKKE---NIPL----------------------------------------------------------------------------------------------------------- : 124
DadjCSP2 : PEGKFAAQFHQFLNA------------------------------------------------------------------------------------------------------------------- : 121
DadjCSP3 : PTGSYKERYLEAKKAE---VAIKPAEDTA----------------------------------------------------------------------------------------------------- : 138
DadjCSP4 : PSASFVKRFETTFESLHGPGRPLESTTPAGNKVDSSEAAGNTIDANITQTSPEGSDGANQPDTTTTAQTITTNPSFSTSTKRPSPIPGLVPFNTFFTNPPIPIRPIVNLNLGGNIGATVSGLFRGLGAIG : 240
DadjCSP6 : PAGSYLTKYSDELRKE---GIVV----------------------------------------------------------------------------------------------------------- : 130
DadjCSP8 : KEGAYKKQYREELKKD---GIKL----------------------------------------------------------------------------------------------------------- : 149
DadjCSP9 : G--------------------------------------------------------------------------------------------------------------------------------- : 115

                    *       280
DadjCSP1 : ------------------------ :   -
DadjCSP2 : ------------------------ :   -
DadjCSP3 : ------------------------ :   -
DadjCSP4 : SRVMDTGAEIAQVVIKSITRPLDL : 264
DadjCSP6 : ------------------------ :   -
DadjCSP8 : ------------------------ :   -
DadjCSP9 : ------------------------ :   -
```

**supplementary Figure S4**. Multiple sequence alignment of *D. adjunctus* CSPs, amino acid sequences were aligned by Clustal Omega and edited using Genodoc. Purple boxes show conserved cysteine residues.

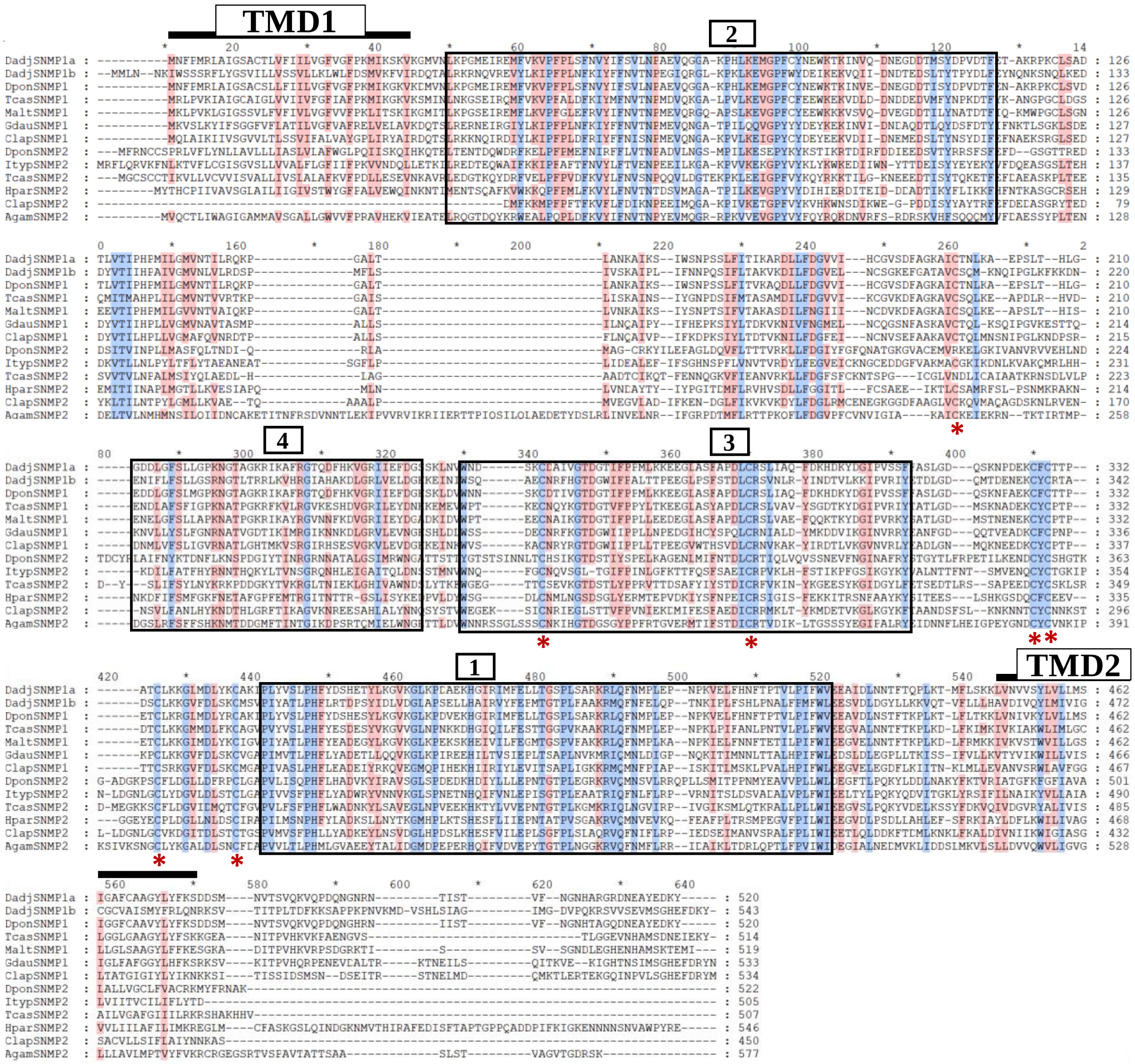


**supplementary Figure S5.** Alignment of both DadjSNMP1 and SNMP of other insect species. Residues with more than 80% similarity are colored in pink and identical residues are colored blue. The asterisks indicate the cysteine residues conserved, the areas in the box below the number 1-4 indicate the four motifs obtained from the MEME analysis, and TMD1/TMD2 shows the transmembrane domain predicted in TMHMM v3.0

**supplementary Table S5.** Protein name and accession numbers used in phylogenetic trees

| OBP | | | | CSP | | SNMP | |
|---|---|---|---|---|---|---|---|
| Number | Acc. No | Number | Acc. No | Number | Acc. No | Number | Acc. No |
| DponOBP2 | N6U2E7 | TcasOBPC11 | D6X4H4 | DponCSP1 | M4W9A4 | DponSNMP1 | M4W9B3 |
| DponOBP2 | M4VUV4 | TcasOBP12 | D2A1G6 | DponCSP1 | A0A0H3W576 | DponSNMP | I1VJ82 |
| DponOBP3 | N6TLD8 | TcasOBPC12 | D6X4H5 | DponCSP2 | A0A0H3W580 | DponSNMP | I1VJ83 |
| DponOBP4 | M4VTH5 | TcasOBP13 | D2A1G5 | DponCSP2 | M4VTH9 | ItypSNMP1 | M3V879 |
| DponOBP6 | M4VTI4 | TcasOBPC13 | D6X4H7 | DponCSP3 | M4VRK2 | TcasSNMP1 | P86905 |
| DponOBP8 | N6T0S9 | TcasOBP14 | D2A254 | DponCSP4 | A0A0H3W5L5 | TcasSNMP01 | D2A0H5 |
| DponOBP8 | M4VRL6 | TcasOBPC14 | D6WPR7 | DponCSP6 | M4VTH1 | TcasSNMP1 | Q7QC49 |
| DponOBP11 | M4W9C1 | TcasOBPC15 | D2A053 | DponCSP6 | A0A0H3W5A7 | HparSNMP1 | A0A2P1ERN0 |
| DponOBP13 | J3JVJ5 | TcasOBP15 | D7ELE9 | DponCSP8 | M4VR62 | AcorSNMP1 | A0A0E3Y6N9 |
| DponOBP16 | N6U049 | TcasOBPC16 | D2A055 | DponCSP11 | M4VUV8 | GdauSNMP1 | A0A4Y5UY78 |
| DponOBP17 | N6UFK3 | TcasOBP16 | D2A1T1 | DponCSPJ19 | I1VJ19 | ClapSNMP1 | A0A310SBT1 |
| DponOBP17 | R9PSQ1 | TcasOBP17 | D2A1G4 | DarmCSP1 | A0A345BT13 | AgamSNMP1 | Q7QC49 |
| DponOBP20 | N6TMW0 | TcasOBP17 | D2A1G3 | DarmCSP2 | A0A3S7PYZ0 | DponSNMP2 | M4VR81 |
| DponOBP20 | M4VUW2 | TcasOBPC17 | D2A056 | DarmCSP3 | A0A345BT14 | DponSNMP2 | N6TNQ9 |
| DponOBP21 | M4VR56 | TcasOBP18 | D2A1G2 | DarmCSP4 | A0A345BT15 | ItypSNMP2 | M3TYZ6 |
| DponOBP22 | M4VRM1 | TcasOBPC18 | D6WAD0 | DarmCSP5 | A0A345BT16 | TcasSNMP2 | Q7Q6R1 |
| DponOBP28 | M4VUX0 | TcasOBPC19 | D6X0T3 | DarmCSP6 | A0A345BT20 | TcasSNMP2 | D6WGA2 |
| DponOBP30 | M4W9B7 | TcasOBPC20 | D6WDX0 | DarmCSP7 | A0A345BT17 | HparSNMP2 | A0A2P1ERQ2 |
| DponOBP31 | M4VRK6 | TcasOBPC21 | D6WRD1 | DarmCSP8 | A0A345BT18 | GdauSNMP2 | A0A4Y5UYF4 |
| DponOBP32 | R9PUL1 | TcasOBP21 | D6WUI5 | DarmCSP9 | A0A345BT19 | ClapSNMP2 | A0A310S638 |

| OBP | | | | CSP | | SNMP | |
|---|---|---|---|---|---|---|---|
| Number | Acc. No | Number | Acc. No | Number | Acc. No | Number | Acc. No |
| DponOBPJ73 | I1VJ73 | TcasOBP22 | D6WYL0 | DvalCSP1 | A0A0H3W576 | AgamSNMP2 | Q7Q6R1 |
| DponOBPJ74 | I1VJ74 | TcasOBP23 | D6X1W0 | DvalCSP2 | A0A0H3W580 | | |
| DponOBPJ75 | I1VJ75 | TcasOBP25 | D6WNH2 | DvalCSP4 | A0A0H3W5L5 | | |
| DponOBPJ76 | I1VJ76 | TcasOBP26 | D6WNH3 | DvalCSP6 | A0A0H3W5A7 | | |
| DponOBPJ77 | I1VJ77 | DarmOBP6 | A0A0X9DLE1 | ItypCSP1 | M3UZC9 | | |
| DponOBPJ78 | I1VJ78 | DarmOBP8 | A0A0X9F313 | ItypCSP4 | M3VHB8 | | |
| DponOBPJ79 | I1VJ79 | DarmOBP13 | A0A0X9FBE8 | ItypCSP5 | M3V872 | | |
| DponOBP6J9 | N6T6J9 | DarmOBP14 | A0A0X9IHE1 | TcasCSP1 | Q0MRL2 | | |
| DvalOBP1 | A0A0H3W5A0 | DarmOBP15 | A0A0X9H1S6 | TcasCSP1 | Q0MRM1 | | |
| DvalOBP2 | A0A0H3W5N4 | ItypOBP2 | M3VHC2 | TcasCSP2 | Q3LB62 | | |
| DvalOBP3 | A0A0H3W572 | ItypOBP3 | M3V875 | TcasCSP10 | D6WT15 | | |
| DvalOBP4 | A0A0H3W574 | ItypOBP5 | M3UZD2 | TcasCSP10 | D6WT15 | | |
| DvalOBP5 | A0A0H3W5L2 | ItypOBP6 | M3VK48 | TcasCSP11 | Q0MRL9 | | |
| DvalOBP6 | A0A0H3W5A2 | Ityp_OBP7 | M3VHC0 | TcasCSP12 | Q0MRL1 | | |
| DvalOBP9 | A0A0H3W5N6 | ItypOBP11 | M3VK50 | TcasCSP12 | Q0MRL6 | | |
| DvalOBP10 | A0A0H3W573 | ItypOBP13 | M3V877 | TcasCSP13 | D6WUE8 | | |
| DvalOBP12 | A0A0H3W577 | ItypOBP14 | M3TYZ1 | TcasCSP13 | D6WUE8 | | |
| DvalOBP15 | A0A0H3W5A3 | ItypOBP15 | M3UZD6 | TcasCSP13 | Q0MRL0 | | |
| DvalOBP16 | A0A0H3W5N8 | TcasOBP1 | D2A670 | TcasCSP14 | Q0MRL8 | | |
| DvalOBP17 | A0A0H3W575 | TcasOBPC01 | D6WS42 | TcasCSP14 | Q0MRK9 | | |
| DvalOBP18 | A0A0H3W579 | TcasOBP2 | D2A673 | TcasCSP15 | Q0MRK8 | | |

| OBP | | | | CSP | | SNMP | |
|---|---|---|---|---|---|---|---|
| Number | Acc. No | Number | Acc. No | Number | Acc. No | Number | Acc. No |
| DvalOBP19 | M4VUX7 | TcasOBPC02 | D6WS43 | TcasCSP16 | Q0MRK7 | | |
| DvalOBP20 | A0A0H3W5A6 | TcasOBPC03 | D6WS44 | TcasCSP16 | Q0MRL5 | | |
| DvalOBP21 | A0A0H3W5P0 | TcasOBP05 | D2A671 | TcasCSP17 | Q0MRK6 | | |
| DarmOBP1 | A0A1D6WII5 | TcasOBPC05 | D6WS39 | TcasCSP17 | D6WCY4 | | |
| DarmOBP2 | A0A1D6WII8 | TcasOBPC06 | D6WS47 | TcasCSP18 | Q0MRK5 | | |
| DarmOBP3 | A0A125QVS5 | TcasOBP6 | D6WM82 | TcasCSP18 | Q0MRK3 | | |
| DarmOBP4 | A0A125QVS6 | TcasOBPC07 | D6WS46 | TcasCSP19 | Q0MRK4 | | |
| DarmOBP5 | A0A0X9F729 | TcasOBP07 | D6WM83 | | | | |
| DarmOBP6 | A0A0X9DLE1 | TcasOBP08 | D6WM81 | | | | |
| DarmOBP8 | A0A0X9F313 | TcasOBPC08 | D6WS45 | | | | |
| DarmOBP13 | A0A0X9FBE8 | TcasOBPC09 | D6WS40 | | | | |
| DarmOBP14 | A0A0X9IHE1 | TcasOBP09 | D6X1W1 | | | | |
| DarmOBP15 | A0A0X9H1S6 | TcasOBP10 | D6WS37 | | | | |
| ItypOBP2 | M3VHC2 | TcasOBPC10 | D6X4H6 | | | | |
| ItypOBP3 | M3V875 | TcasOBP11 | D2A5B1 | | | | |
| ItypOBP5 | M3UZD2 | TcasOBPC11 | D6X4H4 | | | | |
| ItypOBP6 | M3VK48 | TcasOBP12 | D2A1G6 | | | | |
| Ityp_OBP7 | M3VHC0 | TcasOBPC12 | D6X4H5 | | | | |
| ItypOBP11 | M3VK50 | TcasOBP13 | D2A1G5 | | | | |
| ItypOBP13 | M3V877 | TcasOBPC13 | D6X4H7 | | | | |
| ItypOBP14 | M3TYZ1 | TcasOBP14 | D2A254 | | | | |

| OBP | | | | CSP | | SNMP | |
|---|---|---|---|---|---|---|---|
| Number | Acc. No | Number | Acc. No | Number | Acc. No | Number | Acc. No |
| ItypOBP15 | M3UZD6 | TcasOBPC14 | D6WPR7 | | | | |
| TcasOBP1 | D2A670 | TcasOBPC15 | D2A053 | | | | |
| TcasOBPC01 | D6WS42 | TcasOBP15 | D7ELE9 | | | | |
| TcasOBP2 | D2A673 | TcasOBPC16 | D2A055 | | | | |
| TcasOBPC02 | D6WS43 | TcasOBP16 | D2A1T1 | | | | |
| TcasOBPC03 | D6WS44 | TcasOBP17 | D2A1G4 | | | | |
| TcasOBP05 | D2A671 | TcasOBP17 | D2A1G3 | | | | |
| TcasOBPC05 | D6WS39 | TcasOBPC17 | D2A056 | | | | |
| TcasOBPC06 | D6WS47 | TcasOBP18 | D2A1G2 | | | | |
| TcasOBP6 | D6WM82 | TcasOBPC18 | D6WAD0 | | | | |
| TcasOBPC07 | D6WS46 | TcasOBPC19 | D6X0T3 | | | | |
| TcasOBP07 | D6WM83 | TcasOBPC20 | D6WDX0 | | | | |
| TcasOBP08 | D6WM81 | TcasOBPC21 | D6WRD1 | | | | |
| TcasOBPC08 | D6WS45 | TcasOBP21 | D6WUI5 | | | | |
| TcasOBPC09 | D6WS40 | TcasOBP22 | D6WYL0 | | | | |
| TcasOBP09 | D6X1W1 | TcasOBP23 | D6X1W0 | | | | |
| TcasOBP10 | D6WS37 | TcasOBP25 | D6WNH2 | | | | |
| TcasOBPC10 | D6X4H6 | TcasOBP26 | D6WNH3 | | | | |
| TcasOBP11 | D2A5B1 | | | | | | |

Acc. No: Accession number in UniprotKB
